# Cytokine–Ion Channel Interactions in Pulmonary Inflammation

**DOI:** 10.3389/fimmu.2017.01644

**Published:** 2018-01-04

**Authors:** Jürg Hamacher, Yalda Hadizamani, Michèle Borgmann, Markus Mohaupt, Daniela Narcissa Männel, Ueli Moehrlen, Rudolf Lucas, Uz Stammberger

**Affiliations:** ^1^Internal Medicine and Pneumology, Lindenhofspital, Bern, Switzerland; ^2^Internal Medicine V – Pneumology, Allergology, Respiratory and Environmental Medicine, Faculty of Medicine, Saarland University, Saarbrücken, Germany; ^3^Lungen- und Atmungsstiftung Bern, Bern, Switzerland; ^4^Internal Medicine, Sonnenhofspital Bern, Bern, Switzerland; ^5^Faculty of Medicine, Institute of Immunology, University of Regensburg, Regensburg, Germany; ^6^Paediatric Visceral Surgery, Universitäts-Kinderspital Zürich, Zürich, Switzerland; ^7^Department of Pharmacology and Toxicology, Vascular Biology Center, Medical College of Georgia, Augusta, GA, United States; ^8^Novartis Institutes for Biomedical Research, Translational Clinical Oncology, Novartis Pharma AG, Basel, Switzerland

**Keywords:** epithelial sodium channel, Na^+^/K^+^-ATPase, tumor necrosis factor, TNF tip peptide, pneumonia, acute respiratory distress syndrome, lung transplantation, ischemia–reperfusion injury

## Abstract

The lungs conceptually represent a sponge that is interposed in series in the bodies’ systemic circulation to take up oxygen and eliminate carbon dioxide. As such, it matches the huge surface areas of the alveolar epithelium to the pulmonary blood capillaries. The lung’s constant exposure to the exterior necessitates a competent immune system, as evidenced by the association of clinical immunodeficiencies with pulmonary infections. From the *in utero* to the postnatal and adult situation, there is an inherent vital need to manage alveolar fluid reabsorption, be it postnatally, or in case of hydrostatic or permeability edema. Whereas a wealth of literature exists on the physiological basis of fluid and solute reabsorption by ion channels and water pores, only sparse knowledge is available so far on pathological situations, such as in microbial infection, acute lung injury or acute respiratory distress syndrome, and in the pulmonary reimplantation response in transplanted lungs. The aim of this review is to discuss alveolar liquid clearance in a selection of lung injury models, thereby especially focusing on cytokines and mediators that modulate ion channels. Inflammation is characterized by complex and probably time-dependent co-signaling, interactions between the involved cell types, as well as by cell demise and barrier dysfunction, which may not uniquely determine a clinical picture. This review, therefore, aims to give integrative thoughts and wants to foster the unraveling of unmet needs in future research.

## Introduction

Acute lung injury (ALI) and acute respiratory distress syndrome (ARDS) are both clinical syndromes with a high morbidity and mortality rate. Although of a different degree of severity, both ARDS and ALI are characterized by critical gas exchange disturbances, an inflammatory reaction, and an associated alveolar fluid overload (edema). The etiology of ALI and ARDS can be differentiated between direct and indirect lung injury.

The conceptual work presented here discusses the mechanisms regulating alveolar fluid clearance (AFC) during inflammation. As recently demonstrated by several groups, the interaction between cytokines and ion channels may play a critical role in this setting. The presented review does not cover all cytokines and ion channels, but rather focuses on a selection of mainly pre-clinical pathophysiological models and addresses clinical needs and difficulties to effectively translate pre-clinical data into the clinical field. Tables 1–3 give an overview on ion channels and mediator interaction. The ultimate aim of this translational research should be to improve patient care and to reduce morbidity and mortality. This can be achieved by reducing long-term residual sequelae and time on the ventilator, which can improve long-term lung function and health status or health-related quality of life.

**Table 1:** Role of different mediators on fluid transport through impacting on ion channels in the apical and basolateral membrane of epithelial cells.

Channel name	Mediator	Impact on pulmonary barrier function	Mechanism of action
**Apical membrane**			

Epithelial sodium channel (ENaC)	Transforming growth factor beta (TGF-β)	−/+	Decrease in expression during bacterial infection (132) Decreases expression of the αENaC mRNA and protein (132) Internalization of αβγENaC complex from the lung epithelial cell surface and, hence, block the sodium-transporting capacity of alveolar epithelial cells (AECs) (133) Increases the function of ENaC (134)

	Tumor necrosis factor (TNF) receptor binding site	−	Decreases the expression of ENaC mRNA in AECs *in vitro* (135)

	TNF lectin-like domain	+	Activates ENaC (37, 136) Increases ENaC open probability (102)

	Interleukin-1β (IL-1β)	−/+	Decreases the expression of ENaC during bacterial infection (113) Decreases expression of αENaC *via* a p38 MAPK-dependent signaling pathway (113) Suppresses expression of βENaC (137) Decreases ENaC function (138) Augments *in vitro* alveolar epithelial repair (139) Increases ENaC subunits expression in a specific fetal context (140)

	Interleukin-4 (IL-4)	−	Decreases in ENaC expression during bacterial infection (141) Decreases ENaC activity by decreasing the mRNA levels of γENaC and, to a lesser extent, that of the β subunit (142)

	Keratinocyte growth factor (FGF-7)	−	Decreases the expression of αENaC (143)

	Protein kinase C (PKC)	−	Inhibits ENaC function (144–147)

	Cycloheximide (CHX)	−	Downregulate αENaC mRNA abundance similarly *via* the ERK and p38 MAPK pathway (148); Chx effect involves post-transcriptional mechanisms (148)

	Lipopolysaccharide (LPS)	−	Downregulates αENaC mRNA abundance similarly *via* the ERK and p38 MAPK pathways (148); inhibits αENaC promoter activity (148)

	Pneumolysin (PLY)	−	Inhibits ENaC expression upon activation of ERK (102) and inhibits ENaC open probability, by reducing its association with myristoylated alanine-rich C kinase substrate (10, 149)

	Glutathione disulfide (GSSG)	−	Inhibits ENaC activity in primary AECs (150, 151)

	Reactive oxygen species (ROS)	−/+	Inhibit ENaC (150, 152) Decrease channel activity (117) Increases ENaC activity through: (i) Enhancing ENaC gating (153) (ii)Increasing channel abundance (153)

	Ethanol	+	Increases ENaC open-state probability (153) Increases ENaC abundance (153)

	Superoxide (O2^a^)	+	Elevating endogenous (O_2_^–^) levels with a superoxide dismutase inhibitor, prevents NO inhibition of ENaC activity (111)

	Nitric oxide (NO)	−	Inhibits highly selective sodium channels (52, 53)

	Inter-α-inhibitor (IαI)	−	Inhibits ENaC activity in CF patients (154)

	NEDD4-2	−	Decreases the expression of the epithelial ENaC (155)

	Hypoxia	−	Decreases apical expression of ENaC subunits (especially beta and gamma) (156)

	Purinergic receptors (P2YR)	−	Inhibits ENaC expression (157, 158)

	Muscarinic cholinergic	+	Increases ENaC activity. RhoA activity is essential for this process (159)

	Estriadol	+	Increases activity of the non-selective ENaC channels, and these effects are mediated through the G protein-coupled estrogen receptor (160)

	Glucocorticoids	+	Increased in expression of ENaC during bacterial infection (161–164)

	Thyroid hormone	+	Thyroid hormone in concert with glucocorticoids increased the expression of ENaC (165, 166)

	Corticosteroids	+	Increase expression of the γ-ENaC subunit which leads to increase ENaC activity (167)

	Prostasin [channel activating protease 1 (168)]	+	Activates ENaC (169)

	Urokinase-like plasminogen activator	+	Increases the ENaC activity (154, 170–173)

	Cyclic adenosine monophosphate (cAMP)	+	Increases channel activity either by increasing its open probability or by increasing the number of channels at the apical membrane (174)

	Cystic fibrosis transmembrane conductance regulator (CFTR)	+	Activated CFTR can inhibit ENaC (175)

	Dopamine	+	Increases ENaC activity by a cAMP-mediated alternative signaling pathway involving EPAC and Rap1, signaling molecules usually associated with growth-factor-activated receptors (176)

	β2-agonists	+	Activates ENaC (159) Enhancing the insertion of ENaC subunits into the membrane of AECs (156)

	Human AGEs (receptor for advanced glycation end product ligand)	+	Increases ENaC activity through oxidant-mediated signaling (177)

CFTR (Cl^?^ channel)	Interferon-gamma (IFN-γ)	−	Decreases the expression of CFTR mRNA (142, 178)

	TGF-β	−	Decrease CFTR expression and function (179)

	Interleukin-4 (IL-4)	+	Increases the expression and function of CFTR (142)

	Interleukin-13 (IL-13)	+	Increases the CFTR expression (180)

	Interleukin-1β (IL-1β)	+	Increases CFTR expression trough increasing mRNA levels (138, 181)

	β2-agonists	+	β2AR mediates enhancement of AFC *via* increasing Cl? flux through CFTR (182, 183) It activates CFTR by raising cAMP intracellular levels and mediating protein kinase A (PKA) activation (184)

	Na^+^/K^+^ ATPase (Na+/K+-ATPase)	−	Inhibition of the Na+/K+-ATPase lead to a reduced transcription of CFTR (185) CFTR dysfunction occurs through Na+/K+-ATPase inhibition by ouabain (186)

Cyclic nucleotide-gated cation channels (CNG) (Na^+^ channel)	Glucocorticoids	+	Increases mRNA for alphaCNG1 (187)

	mineralocorticoids	+	Increases mRNA for alphaCNG1 (187)

TMEM 16a (CaCC) (Ca+ activated Cl? channel)	CFTR	−	Can inhibit TMEM 16a through attenuation of ionophore-induced rise in Ca^2+^ (188)

	IL-4	+	Increases the expression of CaCC (189)

	IL-9	+	Increases the expression of CaCC (189)

	IL-13	+	Increases the expression of CaCC (189)

ClC-2 (Cl? channel)	TNF	−	Inhibits Aquaporin 5 (AQ-5) Expression (190)

AQ-5 (H_2_O channel)	Transient receptor potential vanilloid 4 (TRPV4)	−	Reduction of AQP5 abundance (191)

	IFN-γ	+	Increases ClC-2 transcripts *via* mRNA stabilization (192)

	cAMP	+	Increasing synthesis of AQP5 mRNA (193) Triggering translocation of AQP5 to the plasma membrane (193)

	Progesterone	+	Increases abundance of AQP5 (194)

	Estradiol	+	Increases in the AQP5 protein level (194)

**Basolateral membrane**			

Na^+^/K^+^ ATPase (Na^+^, K^+^ pump)	IFN-γ	−	Inhibits Na+/K+-ATPase activity (195)

	Interleukin-1β (IL-1β)	+	Increases Na+/K+-ATPase subunit expression (140)

	TNF lectin-like domain	+	Increased Na+/K+-ATPase activity (196) Activation of Na+/K+-ATPase by TIP probably occurs indirectly upon prior activation of ENaC

	TGF-β	−/+	Decrease in Na+/K+-ATPase β1 subunit expression, resulting in decreased Na+/K+-ATPase activity(197, 198) Increases the expression of Na+/K+-ATPase α 1- and β 1-subunits (134)

	TNF-related apoptosis-inducing ligand (TRAIL)	−	Influenza A virus (IAV)-induced reduction of Na+/K+-ATPase is mediated by a host signaling pathway that involves epithelial type I IFN and an IFN-dependent elevation of macrophage TRAIL (199)

	Leukotriene D4	+	Activates Na+/K+-ATPase (200)

	Acetylcholine	+	Activates Na+/K+-ATPase (201)

	NO	−	Inhibits Na+/K+-ATPase (53, 202)

^a^There is growing evidence that ROS are important regulators of ENaC activity and, hence, of epithelial Na^+^ absorption (153). But there is an important question here. Why does ROS increase ENaC activity under some circumstances (e.g., ethanol) but inhibit ENaC under others (e.g., influenza) (153)?

**Table 2:** Impact of different factors on the alveolar–capillary barrier.

Mediator	Impact on pulmonary barrier function	Mechanism of action
**Alveolar epithelium**		
TGFβ1	−	Decreases lung epithelial barrier function (203–205) Increases the permeability of pulmonary endothelial monolayers (206) Increases the permeability of alveolar epithelial monolayers (206)
Tumor necrosis factor (TNF)	−	Causes alveolar epithelial dysfunction (207)

Lectin-like domain of TNF	+	Increases occludin expression, and improved gas–blood barrier function (7)

TNF-related apoptosis-inducing ligand (TRAIL)	−	Disruption of alveolar epithelial barrier (199, 208, 209)

Interleukin-1β (IL-1β)	+	Augments *in vitro* alveolar epithelial repair (139)

Protein kinase D3	−	Dysfunction of airway epithelial barrier through downregulation of a key tight junctional protein claudin-1 (210)

Claudin-3	−	Decreases alveolar epithelial barrier function (211)

Claudin-4	+	Improves the barrier function of pulmonary epithelial barrier by promoting pulmonary fluid–clearance function (211, 212)

Transient receptor potential vanilloid 4 (TRPV4)	−	Disruption of alveolar type I epithelial cells leading to lung vascular leak and alveolar edema (213)

Ethanol	−	Disruption of alveolar epithelial barrier function by activation of macrophage-derived TGFβ1 (214)

Acetoin (butter), diacetyl, pentanedione, maltol (malt), ortho-vanillin (vanilla), coumarin, and cinnamaldehyde	−	Impairment of epithelial barrier function in human bronchial epithelial cells (215)

Asbestos	−	Increases lung epithelial permeability through increasing epithelial fibrinolytic activity (216)

Pneumolysin (PLY)	−	Impairs epithelial barrier (217)

Fas-ligand system	−	Causes alveolar epithelial injury in humans with ALI or ARDS (218) Impairs alveolar epithelial function in mouse lungs by mechanisms involving caspase-dependent apoptosis (219) Inducing apoptosis of cells of the distal pulmonary epithelium during ALI (57)

CO	−	Enhances pulmonary epithelial permeability (220, 221)

**Tight junctions (TJ)**		

Purinergic receptor	+	Preserving integrity of endothelial cell (EC)-cell junctions (222)

Na^+^/K^+^ ATPase	+	Formation of TJs through RhoA GTPase and stress fibers (223) Gene transfer of β1-Na^+^, K^+^-ATPase upregulates TJs formation by enhancing expression of TJ protein zona occludins-1 and occludin and reducing pre-existing increase of lung permeability (224)
	+	

Nitric oxide (NO)	−	Decreases expression and mistargeting of TJ proteins in lung (225)

Influenza A virus (IAV)	−	Disruption epithelial cell TJs (226)

Caveolin-1	+	Regulates the expression of TJ proteins during hyperoxia-induced pulmonary epithelial barrier breakdown (227)

IL-4	−	Causes TJ disassembly and epithelial barrier permeability alteration *via* an EGFR-dependent MAPK/ERK1/2-pathway (228) Reduce protein density at the TJ without causing major changes in cldn1, cldn2, cldn3, and occludin protein levels (229)

IL-13	−	Reduction of protein density at the TJ without causing major changes in cldn1, cldn2, cldn3, and occludin protein levels (229)

TNF	−	Causes TJ permeability (230)

Interferon-gamma (IFN-γ)	−/+	Disorganization of the TJ and an increase in paracellular permeability (231) Promotes epithelial restitution by enhancing barrier function and wound healing (232) It can also reverse IL-4- and IL-13-induced barrier disruption (232)

Trypsin	−	Destroys the TJs which lead to airway leakage

Cigaret smoke	−	Causes disassembly of TJs, modulated through the EGFR–ERK1/2 signaling pathway (233)

Cadmium	−	Causes disruption of TJ integrity in human ALI airway cultures both through occludin hyperphosphorylation *via* kinase activation and by direct disruption of the junction-interacting complex (234)

**Capillary endothelium**		

TGFβ 1	−	Induces endothelial barrier dysfunction *via* Smad2-dependent p38 activation (235)

TNF	−	Disruption of the lung vascular barrier (236, 237) Augmenting endothelial permeability (67, 238) Apoptosis of lung microvascular ECs (39, 239, 240)

Lectin-like domain of TNF	+	Strengthens barrier function or increasing endothelial barrier tightness (9) Protective effect in PLY-Induced endothelial barrier dysfunction (9) Can reduce PLY-induced RhoA/Rac-1 balance impairment and MLC phosphorylation (10) Protects from listeriolysin-induced hyperpermeability in human pulmonary microvascular ECs (241) Reducing vascular permeability (196) Increases in membrane conductance in primary lung microvascular ECs (242)

IFN-γ	−	Increases vascular permeability (243)

Interleukin-1β (IL-1β)	−	Given intratracheally, IL-1β increased endothelial permeability and lung leak (244–247) Increases vascular permeability (243)

Interleukin-2 (IL-2)	−	Increases vascular permeability (248)

Interleukin-6 (IL-6)	−	Increases endothelial permeability (249)

Interleukin-8 (IL-8)	−	Increases endothelial permeability (250)

Interleukin -12 (IL-12)	−	Upregulate the release of the vascular permeability factor which is a lymphokine derived from LN peripheral blood mononuclear cells (251)

Neutrophils	−	Inducing endothelial barrier disruption through secretion of leukotrienes or heparin-binding protein, direct signaling into the EC *via* adhesion-dependent mechanisms and production of ROS (252)

ENaC	+	ENaC-α can strengthen capillary barrier function (9)

TRPV4	−	Increases in vascular permeability thus promoting protein and fluid leak (253) Applying TRPV4 inhibitors exhibits vasculoprotective effects, inhibiting vascular leakage, and improving blood oxygenation (254)

Thrombin	−	Increase in endothelial permeability (255)

Platelet-activating factor	−	Increase in endothelial permeability (256)

Hydrogen peroxide	−	Increase vascular permeability through enhancing vascular endothelial growth factor expression (257)

Integrin αvβ5	−	Increases pulmonary vascular permeability (258)

T-cadherin	−	Causes enhancement of endothelial permeability (259)

Myosin light chain kinase	−	Vascular hyperpermeability (260)

Lipopolysaccharide (LPS)	−	Induces lung endothelial barrier dysfunction (261)

PLY	−	Impairs endothelial barrier (10, 262)

P2Y receptors	+	Regulators of lung endothelial barrier integrity (263)

CO	−	Enhances pulmonary epithelial permeability (221)

Soluble receptor for advanced glycation end products	−	Increase in alveolar–capillary barrier permeability (264)

**EC adhesion**		

Podocalyxin	+	Decreases vascular permeability of ECs by altering EC adhesion (265)

NLRP3	+	Protects alveolar barrier integrity by an inflammasome-independent increase of epithelial cell adherence (266)

**Table 3:** Comparison of the properties of highly selective and non-selective channels.

	Highly Selective	Non-selective
Na/K selectivity (267, 268)	>40	1.1
Unit conductance, pS (267–269)	6	21
Amiloride K_i_, nM (268, 270)	38	2,300
Increased cellular cyclic adenosine monophosphate or β-adrenergic stimulation (271, 272)	Channel surface density increases	P_o_ increases
Increased cGMP or NO (273)	P_o_ decreases	P_o_ decreases
Protein kinase C activation (274, 275)	P_o_ decreases, surface density decreases	Channel surface density increases
Increased intracellular Ca^2+^ (271)	No effect	P_o_ increases
Purinergic stimulation (276–279)	P_o_ decreases	P_o_ increases
Dopaminergic stimulation (176, 280)	P_o_ increases	No effect
Superoxide production (111)	P_o_ increases	Channel surface density increases
Hypoxia (268)	Channel surface density decreases	Channel surface density increases

*P_o_, channel open probability; K_i_, inhibitory constant, i.e., the dose that reduces open probability by 50%*.

The main task of the lungs is to account for the efficient external gas exchange between air and the blood. Only a thin barrier of several micrometers separates the pulmonary capillaries from the immense alveolar surface, mainly made up by alveolar type I cells. An intimately fine, deformable, tensible, flexible, and continuous net of interstitial tissue integrates the interstitial net around vessels and bronchi. The whole system has to be “breathable,” i.e., has to be efficiently moved by the thoracic cage to transport fresh air in the alveolar space that matches to the vascular bed for gas exchange. A number of structural and physiological features prevent alveolar flooding. These protective mechanisms include the very low vascular resistance in the pulmonary circulation, the high capillary colloid-osmotic pressure and, on the other hand, the diminished interstitial colloid-osmotic pressure in case of increased filtration. The minimal mechanic stress of alveolar septa due to surface tension reduction by surfactant as well as the optimal active fluid reabsorption out of the alveolar space are further measures that optimize fluid clearance. Structurally, a rather tight pulmonary microvascular endothelium allows for a minimal continuous filtration of water, micro-and macromolecules, with an even tighter alveolar epithelium (1). All three fluid compartments, the capillaries, the interstitium, and the alveoli are in a complex dynamic equilibrium. The continuous pulmonary interstitial space is a drainable continuum that is ultimately emptied by the lymphatic vessels. There is a basal transendothelial filtration of about 10 ml/h that increases up to tenfold during physical activity. When such filtered fluid enters the alveolar interstitial space, it moves proximally to the peri-bronchovascular space (2). Under normal conditions, most of this filtered fluid will be removed by the lymphatics from the interstitium and returns to the systemic circulation (2).

The interstitial compartment is a reversible store of excess fluid. In the adult lung, interstitial fluid—or interstitial edema—can mount up to a volume of 500 ml. However, at that volume there is usually already some alveolar edema (3). It was formerly wrongfully postulated that the Starling filtration forces, which essentially represent the balance between oncotic and hydrostatic pressures in the capillaries and the interstitial space, are the only driving forces for liquid flow from the bloodstream into the extravascular space. In the last four decades, four important refinements have been made. The first one is that fluid reabsorption from the alveolar space is mainly performed by active vectorial Na^+^ transport (4). Moreover, also Cl^−^ transport was suggested to be important, leading to consecutive counter-ion transport, as well as to an osmotic water shift. In the last few years, a second refinement has been made which mainly occurs in heart failure, namely that pumps which usually free the alveolus of ions can also provide inverse transport (5). This biological “emergency plan” in case of hydrostatic pulmonary edema widens the scope of mechanisms in cardiogenic lung edema, as one can argue that in heart failure these mechanisms could be rescue fluid shifts including into the alveolar space, and that a concerted fluid management in vascular, renal, and intestinal and pulmonary vascular beds might occur in severe cardiac failure or fluid overload, taking into account some degree of alveolar pulmonary edema. A third rather novel field is the research on emptying of the alveolar space from its protein load; but so far only few insights in this clinical topic exist (6). The fourth refinement is the close relationship of ion channel activation with barrier tightness. Interactions between the lectin-like domain of tumor necrosis factor (TNF), mimicked by its amino acid-identic TNF tip peptide (a.k.a. AP301 and Solnatide) and the epithelial sodium channel (ENaC) were shown to have a clear effect on epithelial (7) and endothelial barrier tightness (8, 9). As such, ion channel activity and barrier tightness may be key survival factors for tissue function, be it the lung or the kidney, the brain or other organs, and for tissue stability (9–11).

Alveolar fluid reabsorption is a very physiological process that is even required directly after birth where the lung has to be cleared from liquid as it has been so far immersed in the amnionic fluid. In premature infant, insufficient clearance of lung liquid at birth may lead to respiratory distress syndrome (RDS). The key clinical relevance of the physiological role of αENaC in the lungs has been confirmed in the mouse in which the ENaC-α gene was deleted by a homologous recombination. These animals were not able to remove alveolar fluid from their lungs and died shortly after birth (12). Surprisingly, in humans this situation seems more complex, as a child with an inactive homozygous ENaC-α mutation did not suffer perinatal respiratory failure (13).

**Figure 1:**
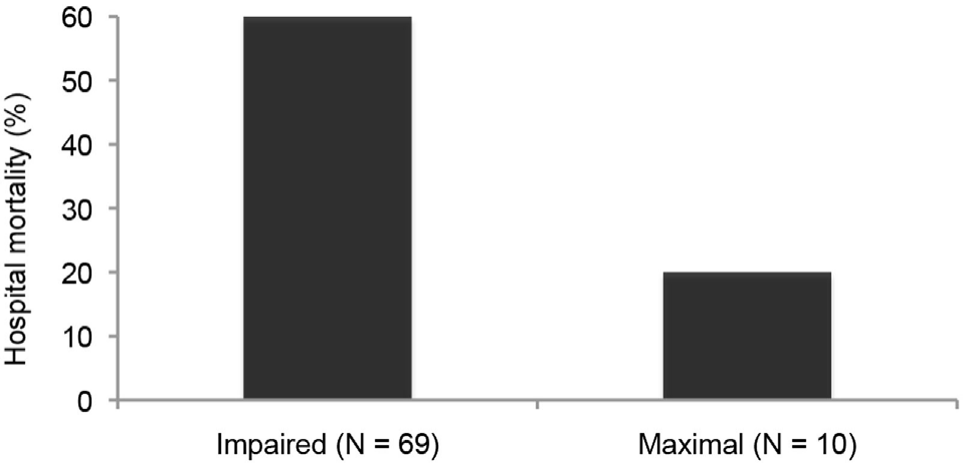
Hospital mortality is increased in patients with acute lung injury or the acute respiratory distress syndrome with impaired fluid clearance (17).

Likewise in adults with heart failure or RDS, while they show no active fluid clearance greater morbidity and mortality rate is probable (14). In clinical studies using quantification of protein in alveolar liquid, prognosis was dependent on the estimated AFC. In a recent study, 56% had impaired AFC, and only 13% a maximal AFC rate (Figure 1). Survival was higher and days on mechanical ventilation were less in those patients with maximal alveolar clearance rate compared to patients with impaired clearance rate. With hydrostatic edema, by contrast, 75% of patients had submaximal to maximal AFC (15). Of note is that in hydrostatic edema alveolar fluid shift may even actively be reversed (5, 16) as discussed above.

## Pulmonary Edema

Pulmonary alveolar edema is a life-threatening state that results from an imbalance between passive and active forces driving fluid into the airspaces and those mechanisms involved in its removal (1, 4). Based on the underlying cause, in the next two chapters we will discuss two main fundamentally different types of pulmonary edema occur in humans (2).

### Cardiogenic or Hydrostatic Edema

**Figure 2:**
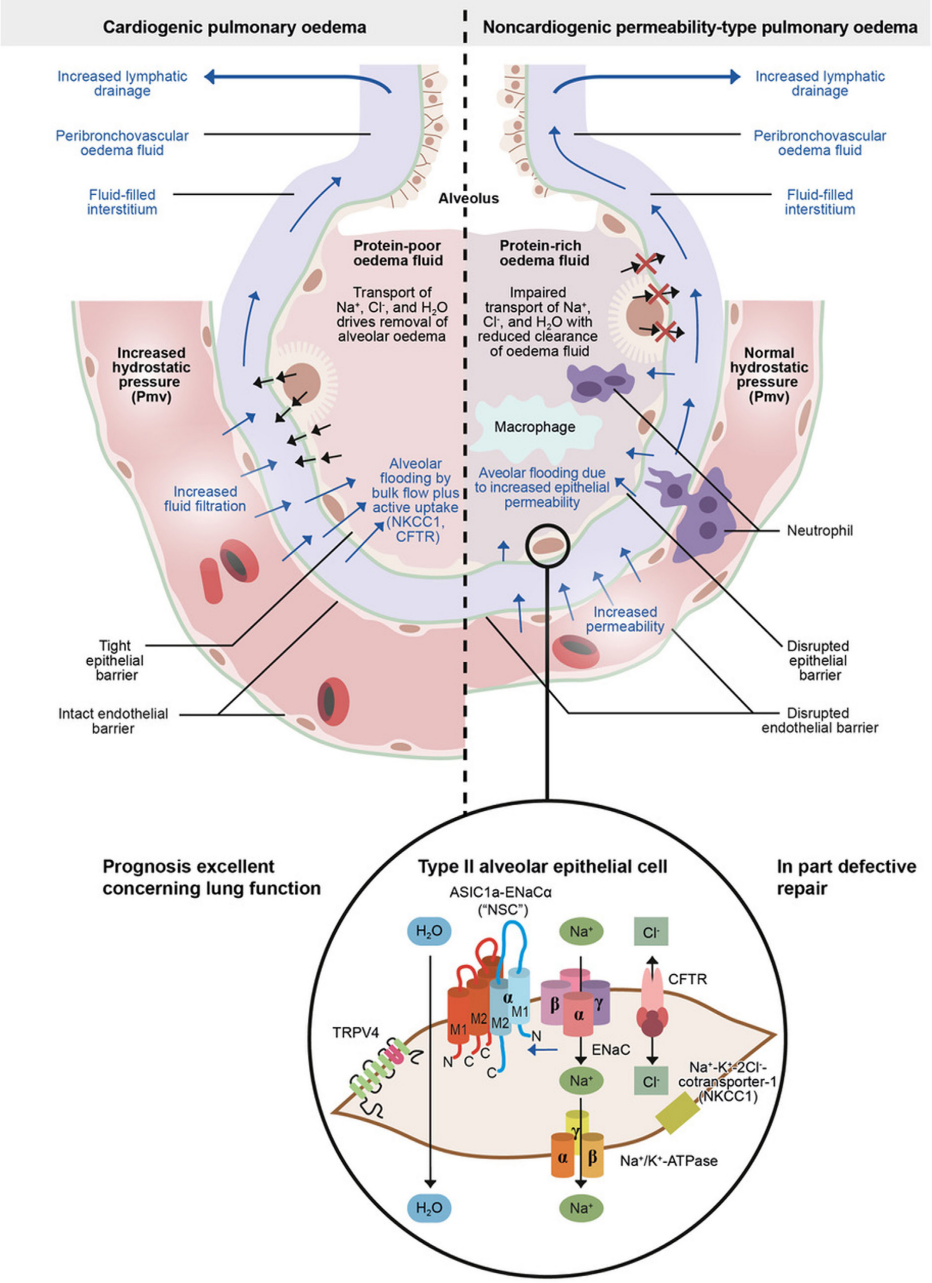
Physiology of microvascular fluid exchange in the lung.

Cardiogenic pulmonary edema (also called hydrostatic or hemodynamic edema) (2) is caused by an increased capillary hydrostatic pressure, secondary to an elevated pulmonary venous pressure (18) (Figure 2, left panel). This type of edema can occur following left ventricular heart failure, renal failure, or fluid overload, or arteriovenous shunts or fistulas. Left heart failure is most commonly caused by myocardial ischemia with or without myocardial infarction, exacerbation of chronic systolic or diastolic heart failure, or dysfunction of the mitral or aortic valve. Acute cardiogenic pulmonary edema is a frequent medical emergency that accounts for up to 1 million hospital admissions per year in the United States and for about 6.5 million hospital days each year, and is typically present during acute cardiac failure in 75–80% of patients (19). Coronary heart disease may account for about half to two-thirds of heart failures. There has been an increase in cardiac failure patients as well as in hospitalization rate during the last decade (20). As a matter of fact, heart failure is the most rapidly growing cardiovascular condition globally. The reported Western world life time risk is typically about 33% for men and 29% for women for our population, and depends, besides sex, on comorbidities and cardiovascular risk factors, such as arterial hypertension, diabetes, obesity, sleep related disorders, smoking, sedentary lifestyle, and ethnic background (20). In patients aged 65 years and older, more than 10% suffer from congestive heart failure (21). Interstitial pulmonary edema and alveolar flooding impair lung mechanics and gas exchange, thus causing dyspnea and tachypnea, which ultimately results in an age-dependent in-hospital mortality rate of about 15% (22).

The development of pulmonary edema is characterized by increased transcapillary hydrostatic pressure gradients. Moreover, a reversed and active electrolyte flow and its resulting active fluid transport can be involved (5, 23). This is possible by the bidirectional permeation permitting anion channels cystic fibrosis transmembrane conductance regulator (CFTR) and NKCC1 (16), which seems to account for up to 70% of the total alveolar fluid influx at elevated hydrostatic pressure. It is supporting the concept that alveolar fluid secretion is a secondary consequence of impaired alveolar Na^+^ uptake (16). Both CFTR and NKCC1 are inhibited by furosemide. This might explain why in the clinical heart failure setting furosemide immediately relieves patients, i.e., by inhibition ion and, thus, fluid transport into the alveolus during alveolar lung edema generation when furosemide is administered, and not only after a huger delay of about half an hour or more when the renal effect of relevant diuresis has occurred. However, also a venous vasodilation, direcly reducing preload, occurs immediately after systemic furosemide administration (24).

A rapid increase in hydrostatic pressure in the pulmonary capillaries, leading to increased transvascular fluid filtration, and even active fluid transport as mentioned above, is the sign of acute cardiogenic or volume-overload edema (Figure 2, left panel). Such an increase could be usually due to elevated pulmonary venous pressure from increased left ventricular end-diastolic pressure and left atrial pressure (2). Mild elevations of left atrial pressure (18–25 mmHg) cause edema in the peri-microvascular and peri-bronchovascular interstitial spaces (1). Excess interstitial fluid is transported by lung lymphatics into the vascular system. A negative interstitial pressure gradient, even under conditions of edema, is the major force for the removal of pulmonary interstitial edema fluid into the lymphatics (25). If left atrial pressure rises further (>25 mmHg), edema fluid passes through the lung epithelium, in part by active transport, flooding the alveolar space with protein-poor fluid (Figure 2, left panel) (1, 2, 5). By contrast, non-cardiogenic pulmonary edema is based on increased pulmonary vascular permeability, resulting in an increased flux of fluid and macromolecules into the pulmonary interstitium and airspaces (Figure 2, right panel) (2).

There is a considerable link between inflammation and heart failure. The Val-HeFT study demonstrated a direct correlation between elevated levels of C-reactive protein and heart failure severity, and C-reactive protein predicts the risk of death and early readmission in acutely decompensated heart failure (26). As reviewed by Azzam et al. in this topic issue, one hypothesis is that heart failure is accompanied by systemic and mesenteric venous congestion, which may in turn cause bowel edema and a consecutive increased permeability, leading to bacterial translocation, endotoxin release, and resultant systemic inflammation. A second hypothesis postulates that the failing, but not the healthy, heart has the ability to produce pro-inflammatory TNF during dilated myopathy. Third, decreased cardiac output could cause systemic tissue hypoxia with subsequent systemic inflammation, which might be the primary stimulus for increased TNF production (21).

Soluble TNF receptor-1 and interleukin-8 (IL-8) are independently associated with cardiovascular mortality, as is endothelin-1. In transgenic mice overexpressing TNF the left ventricular ejection fraction was depressed depending on TNF gene dosage (21). TNF has been associated with worsened prognosis. However, two studies aiming to neutralize the cytokine in heart failure, using the soluble human TNF receptor 2 construct etanercept, were stopped because of lack of clinical benefit and patients receiving the highest dose even had increased adverse outcomes (27). Similar results were observed with the neutralizing antibody infliximab (28). Whether the negative results are explained by inappropriate blocking of a “physiological” inflammation linked with tissue-reparative processes such as cardiac remodeling, or whether other mechanisms like too advanced heart failure, infections, toxicity of treatment, or genetic polymorphisms are involved, remains open, and should be further studied (21). Recently, it was suggested that beneficial or detrimental effects of TNF neutralizing agents depend on whether they spared or rather blunted discrete amounts of TNF that preconditioned cardiomyocytes to make them more resistant to high concentrations of the cytokine (29). The results, however, put forward that cytokines are effectors and not solely biomarkers in heart failure. Furthermore, reparative processes in the myocardium are accompanied by reactive or replacement fibrosis, mediated by TGF-β1, endothelin-1, and angiotensin-II (21). Angiotensin-II decreases AFC *via* cyclic adenosine monophosphate (cAMP) effect on the Na^+^/K^+^-ATPase pathway. It is involved through p38 and possibly p42/44 MAP kinases with myocardial hypertrophy, inflammation, and neurotransmitter and catecholamine synthesis and release in the brain. Angiotensin-II regulates the NF-κB-dependent gene expression in response to IL-1β stimulation by controlling the duration of ERK and NF-κB activation (21). Many immune cell functions are moreover coupled to intracellular pH. As such, a higher pH represents an important signal for cytokine and chemokine release, and a low pH can induce an efficient antigen presentation. The pH regulating Na^+^/H^+^ exchanger isoforms may play a role in these events (30).

The kidney is a major target organ and a modulator in the pathogenesis of heart failure at least partially by means of the renin–angiotensin system. In initial heart failure, it aims at blood pressure maintenance by direct systemic vasoconstriction, *via* augmentation of the sympathetic nervous system activity and by promoting renal Na^+^ retention. The latter mechanism is deleterious in the progress of cardiac failure and is characterized by enhanced Na^+^ reabsorption in the proximal tubule and collecting duct induced by effects of angiotensin-II and aldosterone on NHE3 and ENaC, respectively (21). Two-thirds of filtered Na^+^ is reabsorbed in the proximal tubule *via* transporters for amino acids, glucose, phosphate and *via* NHE3. At the distal tubule, Na^+^ is reabsorbed by Na^+^, K^+^ co-transporter, which is sensitive to thiazide. In the collecting ducts, a minimal amount of sodium is reabsorbed by ENaC and this is increased by aldosterone. The counterbalance by the natriuretic and vasodilatory atrial natriuretic peptide is dominated at that point by angiotensin-II and aldosterone effects, attenuates endothelial-dependent renal vasodilation and leads to endothelial dysfunction characteristic of cardiac heart failure (21). Heart failure also causes a vasopressin-dependent water reabsorption which maintains blood pressure in the failing heart and further increases fluid retention. The renin–angiotensin system, especially angiotensin-II, activates the immune system and *vice versa*. TNF and IL-6 stimulate the generation of angiotensinogen, exaggerate sodium retention and enhance renal fibrosis. Angiotensin-II enhances TNF and IL-6 in cardiomyocytes and in renal cortical and tubular cells, impairs mitochondrial function, and is pro-oxidative (21). CRP also directly activates endothelin and by this may potentiate a pulmonary vasoconstriction. The review by Azzam et al. in this issue further discusses the causative role of cytokines in the development of cardiogenic edema.

### Non-Cardiogenic or Permeability Pulmonary Edema

Non-cardiogenic pulmonary edema, also known as permeability pulmonary edema, accompanies ALI, pneumonia, pulmonary reimplantation response after lung transplantation, or ARDS (2, 31) (Figure 2, right panel). During the course of these diseases, the interstitium and the alevolus are sites of intense inflammation by an innate immune cell-mediated damage of the alveolar endothelial and alveolar epithelial barrier, with consecutive exudation of protein-rich pulmonary edema fluid (31–33), as recently reviewed by Thompson et al. (31).

This type of pulmonary edema occurs due to modifications in barrier function of the pulmonary capillary or alveolar epithelial compartments as a consequence of either a direct or an indirect pathological process (31). There is some evidence that direct injury, such as pneumonia, aspiration, or pulmonary contusion, mainly affects epithelial barriers, whereas indirect blood-borne insults such as severe sepsis, non-thoracic trauma, pancreatitis, or burns may predominantly target the capillary endothelium (34). Permeability edema accompanies a spectrum of illnesses, ranging from the less severe form of ALI to ARDS (18). Variations in histology and in fluid management strategies suggest different ARDS subphenotypes (31). Apart from ARDS, ALI and severe pneumonia, also lung transplantation can be accompanied by acute pulmonary edema by the pulmonary reimplantation response (35). Ischemic vascular injury of the allograft results in increased permeability of the lung after reperfusion and in turn leads to interstitial and alveolar edema (33).

**Figure 3:**
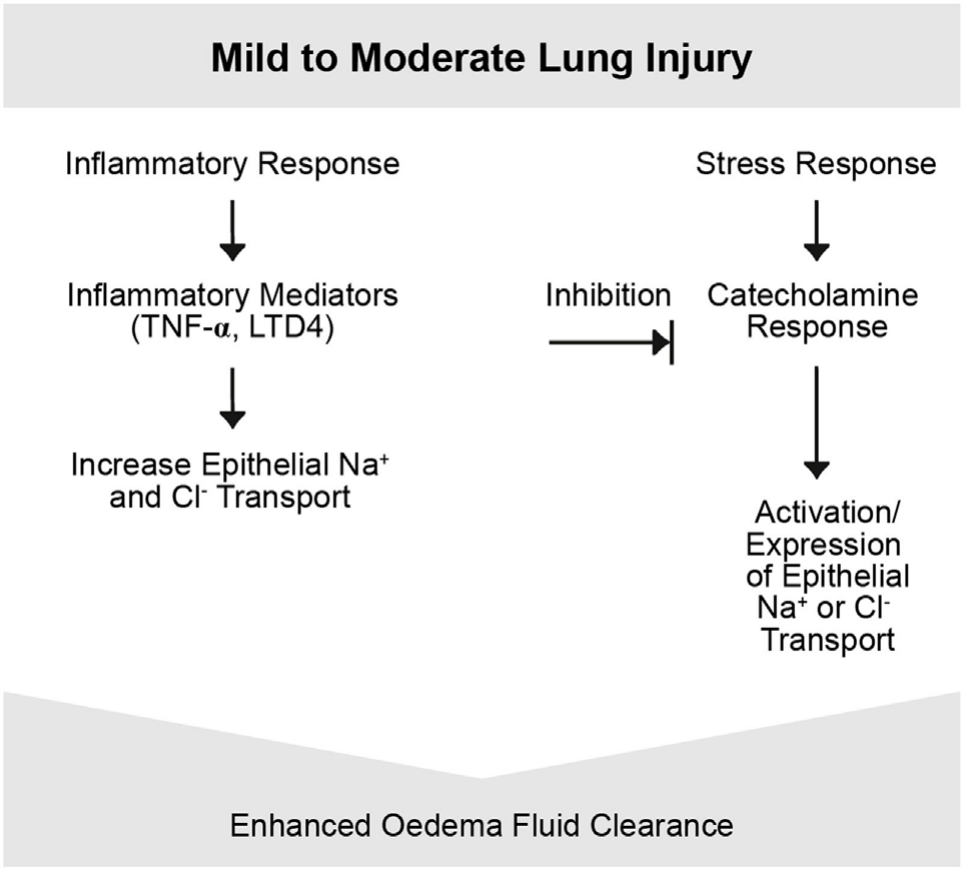
Mild-to-moderate lung injury. Mild-to-moderate lung injury may lead to enhanced edema clearance. This response is due to an activation of epithelial Na^+^ transport probably based on the increased endogenous catecholamine production associated with the insult. However, in certain types of injury, other pathways may be involved. Other inflammatory mediators such as tumor necrosis factor (TNF) potentially participate (4).

**Figure 4:**
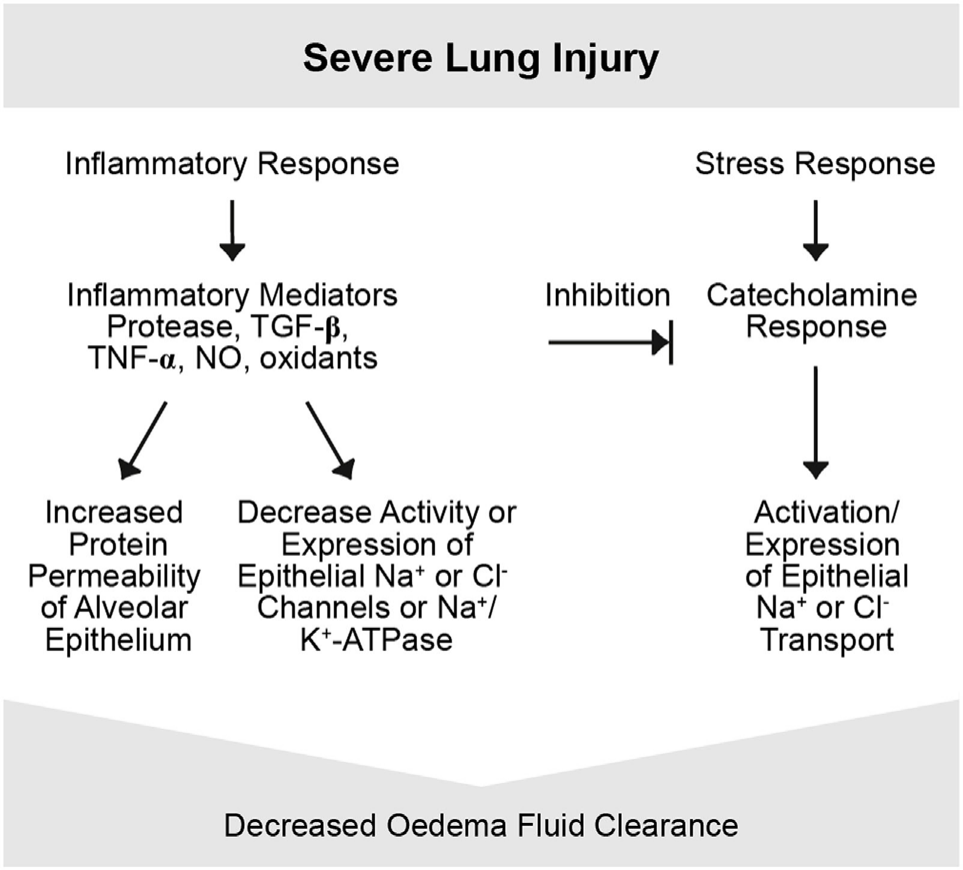
Severe lung injury. Severe lung injury may usually lead to decreased edema clearance. Severe injury usually includes alveolar epithelial injury and, thus, increases epithelial permeability and electrolytes and is associated with reduced epithelial Na^+^ transport. Inflammatory mediators are involved in this response, such as proteases, tumor necrosis factor (TNF), TGF-β, nitric oxide (NO), and oxidants. Possibly the intensity of the inflammatory response may transform a mild to a severe lung injury form by inducing changes in function and integrity of the alveolar epithelium and endothelium (4).

The extent of alveolar edema depends on the competing effects of increased permeability and the active edema fluid clearance from the alveolar space in regions where the epithelium is undamaged (31, 36). Inflammation plays a key role in the pathogenesis of permeability edema (37, 38) and can lead to the orchestration of a great variety of inflammatory and non-inflammatory cells, the former of which can locally release pro-inflammatory mediators such as TNF, LTD4 (32). There may also be endothelial and alveolar epithelial cell (AECs) death, which can further contribute to organ dysfunction and leak (39, 40). Moreover, a cascade of inflammation and a downregulation of repair mechanisms may occur (Figures 3, 4).

Cells of the innate immune system, such as activated alveolar macrophages and recruited polymorphonuclear granulocytes (PMN) and also cells from the adaptive immune system, such as T_H_17 cells can interact in ALI and ARDS and release huge amounts of mediators (31). Thrombo-coagulative processes ensue, e.g., TNF-mediated by tissue factor, with a proaggregatory role for platelets. Preventive aspirin was recently shown to protect from ARDS (41). Regional tissue overdistension especially during ventilation and repetitive opening and closing of inflamed alveolar spaces amplify the regional inflammation, further denaturing surfactant, underlining the vital importance of protective ventilation strategies and positions.

Although pulmonary edema is one of the most frequent medical emergencies, clinically it is sometimes difficult to differentiate between its two main subtypes: cardiogenic and non-cardiogenic edema (2). Moreover, to date, no proven drug therapy is available for permeability edema associated with ALI and ARDS (2, 31, 38). Morbidity and mortality inversely correlate with AFC capacity in this setting (42, 43). The severity of shock in sepsis-induced ARDS is associated with lower AFC (44).

As mentioned above, 56% of patients with permeability pulmonary had an impaired AFC, and only 13% a maximal AFC rate (Figure 1). Survival of patients with maximal alveolar clearance rate was higher, as compared to patients with abnormal clearance rate, and the days on mechanical ventilation was less in this group. Clinically impressive is also a series of post-lung transplant patients showing a relation between total ischemic time and the degree of post-transplantation protein-rich and highly neutrophil-rich (71–99% of cells) permeability edema. Those patients with the best AFC had the best clinical outcomes, including the least and the fastest resolving pulmonary reimplantation response (45). Thus, the ability to reabsorb fluid from the alveolar space was a marker of less severe reperfusion injury. These findings indicate that intact alveolar epithelial fluid transport is critically important for a timely recovery from post-transplantation reperfusion pulmonary edema.

## Pulmonary Fluid Balance through Barriers

Airways normally have a critically regulated fluid layer essential for normal gas exchange and removal of foreign particulates from the airway. Maintaining this fluid layer in the alveoli also depends critically on sodium reabsorption. The pulmonary epithelium serves as a barrier to prevent access of the inspired luminal contents to the subepithelium (11) and modulates the initial responses of the airways and lung to both infectious and non-infectious stimuli (11). One mechanism by which the epithelium achieves this is by coordinating transport of diffusible molecules across the epithelial barrier, both through and between cells (11). Specific elements of pulmonary alveoli play different roles as a barrier maintaining the pulmonary fluid balance (38). These barriers will be discussed in more detail below.

### Epithelial Barrier

Lung epithelium is a mucosal surface composed of ciliated cells, mucus-producing cells, and undifferentiated basal and progenitor cells. This dynamic barrier forms the interface between the lumen and the parenchyma from the upper airways to the alveoli. The lung epithelium constantly responds to luminal stimuli and coordinates its response to maintain homeostasis in the lung (11). A breakdown in this coordinated response can cause different lung diseases (11). The alveolar epithelium (0.1–0.2 µm) covers 99% of the airspace surface area in the lung (46) and contains a number of important cell types. Type I cells (AT1) cover at least 95% of the alveolar surface and are the apposition between the alveolar epithelium and the vascular endothelium. This provides a tight barrier that facilitates efficient gas exchange and which is involved in fluid and protein movement from the interstitial and vascular sites (38, 47) and its reabsorption *vice versa* (4, 5). The role of aquaporin 5 (AQ-5) in AFC is not clear, in view of the normal AFC capacity in physiological situations in AQ-5 knock out mice (48). The osmotic clearance of water secondary to the ion transport gradient across the alveolar epithelium probably occurs by paracellular pathways and not by the assumed transcellular using aquaporin 5 (25); however, their role in injury is not fully excluded (4). Type II cells (AT2) cover about 5% of the alveolar surface and are known especially for their key function in surfactant secretion and in vectorial transport of Na^+^ (49), a major driving force for fluid removal from the alveolar space. Amiloride-sensitive sodium channels on the apical, “air-faced,” surface, mainly the ENaC, are key channels in alveolar fluid transport (50, 51), with the driving force stemming from the Na^+^/K^−^-ATPase on the basolateral, “blood-faced,” surface (46). Dysfunction of these Na^+^ transporters during inflammation can contribute to pulmonary edema (52–54). Tight junctions (TJ) that connect adjacent epithelial cells near their apical surfaces and maintain apical and basolateral cell polarity are fundamental to create a permeability barrier required to preserve distinct compartments in the lung (55).

Alveolar and distal airway epithelia are surprisingly resistant to injury, particularly if compared to the adjacent lung endothelium. When lung endothelium gets injured, the alveolar epithelial barrier may retain its normal impermeability and its normal fluid transport capacity, as seen in animal models with LPS given intravenously or intratracheally (4). This might explain why in mild-to-moderate lung injury AFC may not only be preserved, but even upregulated by stress hormones—an effect that may be inhibited by amiloride or propranolol.

However, in severe ALI, ARDS, and pneumonia, epithelial cell death may occur, as has been shown in a seminal morphological study published 4 decades ago by Bachofen and Weibel (56). A central role for soluble Fas ligand (FasL) has been proposed in AT1 and AT2 cell death, and an association between its levels in bronchoalveolar lavage level on day 1 of ARDS and patient death has been proposed (57, 58). However, there may be extensive crosstalk between injurious, inflammatory, and death cascades and repair in the lungs, as well as in other organs in patients with ARDS. Direct alveolar cell death may probably also occur due to bacterial exotoxins or stresses like overdistension. Such epithelial cell death may make the lungs prone to increased permeability and thus disturb AFC, as well as to the danger of disordered repair, such as in fibroproliferative ARDS.

Recent work on different predictors of ARDS suggests that the degree of AT1 cell injury is a central determinant of outcome in ALI and ARDS. Receptor for advanced glycation end products (RAGE) is an immunoglobulin superfamily member, involved in propagating inflammation. RAGE is abundant in the lungs and can be primarily found in AT1 cells. Higher baseline plasma levels of RAGE were found to be associated with worse outcome, including less ventilator-free days and increased mortality, and it excellently discriminated in sepsis patients for the diagnosis of ARDS. Higher levels in bronchoalveolar lavage also predicted post-lung-transplant primary graft failure and correlated with its grade of severity (59). Apart from RAGE, also surfactant protein D level, an AT2 cell product, was, together with the neutrophil chemokine IL-8 (CXCL8), the best performing biomarker for poorer outcome in terms of mortality (60).

### Endothelial Barrier

The capillary endothelial barrier also functions as a key component to maintain the integrity of the vascular boundaries in the lung. The gas exchange surface area of the alveolar–capillary membrane is extremely huge and optimized to facilitate perfusion–ventilation matching (61). Pulmonary endothelium separates also the intravascular marginated pool of polymorphonuclear neutrophils from the airspaces. The endothelium, the most abundant cell relative to the total cell population in the lung, has additional key regulatory roles apart from gas exchange, namely vascular tone *via* nitric oxide (NO) and endothelin-1, and coagulation, as recently discussed in depth in a review on the endothelium and ARDS (34).

In the pulmonary microvasculature, the endothelial cells (ECs) form a semi-permeable barrier between the blood and the lung interstitium (38). Disruption of this barrier may occur during inflammatory disease such as pneumonia, ALI, ARDS, or ischemia–reperfusion injury. In sepsis, early microcirculatory perfusion indices are more markedly impaired in non-survivors, as compared to survivors and correlate with increasing severity of vascular dysfunction (62). Lung ECs are considered orchestrators of the inflammatory response. These cells can directly sense pathogens *via* toll-like receptors and may contain local bacterial spreading by coagulation, leading to capillary thrombosis and extravascular fibrin deposition (34). This contributes to an increased dead-space fraction that correlates with clinical outcome (63). In sepsis, overwhelming EC activation can lead to apoptosis within minutes to hours (64), which in turn increases barrier permeability and subsequent mortality (65). In ARDS, EC death can occur in by mechanical insults, like shear stress, and by pro-inflammatory mediators, including TNF, angiostatin, and TGF-β (39).

**Figure 5:**
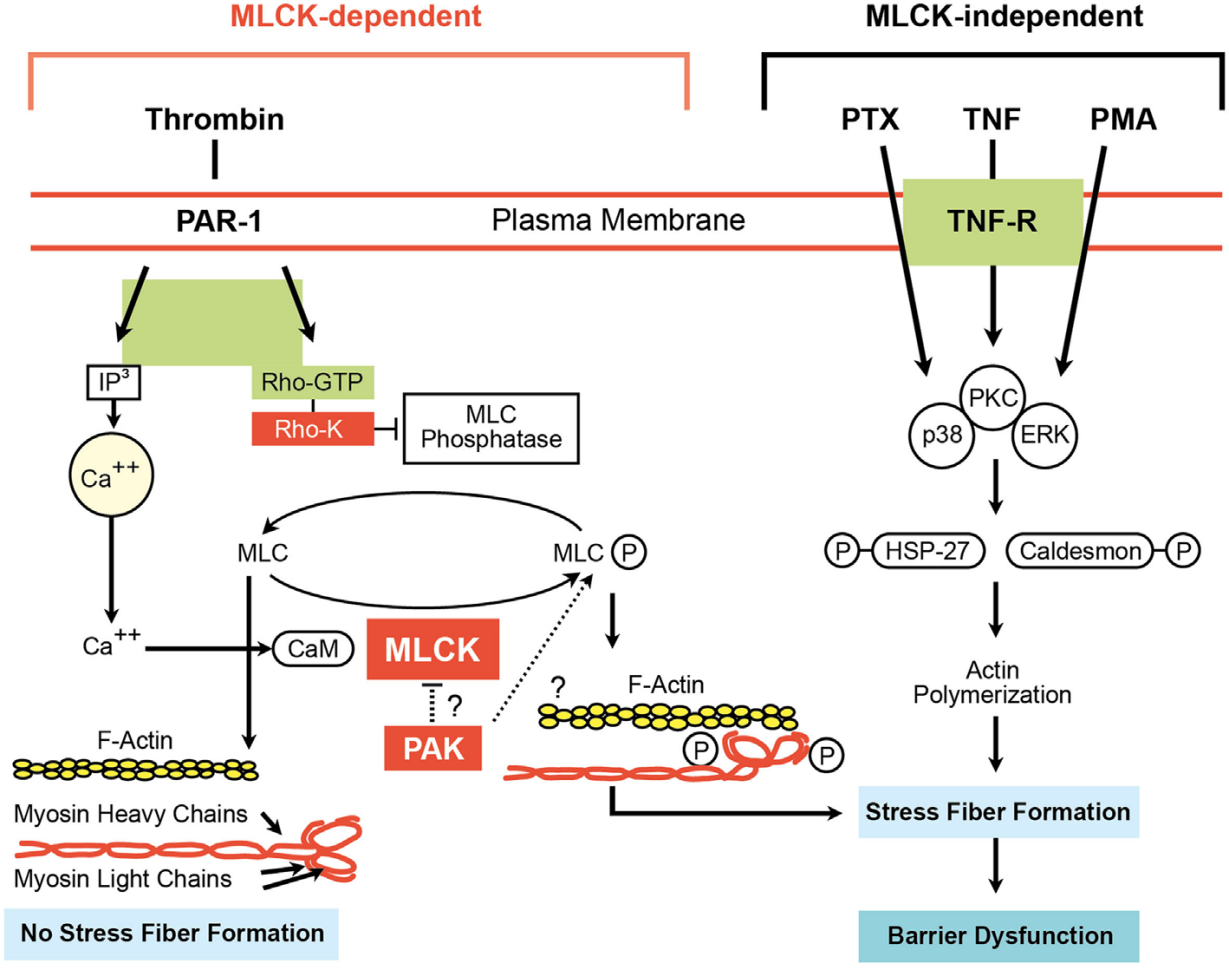
Myosin light chain kinase (MLCK)-dependent and MLCK-independent pathways involved in endothelial cell (EC) barrier dysfunction (66). Adapted from Ware and Matthay (2).

Intercellular junctions act as dynamic structures and do not statically resist entry to all substances. They that can open or close in response to physiological or pathological stimuli. Figure 5 presents some potential pathways regulating EC barrier function (66). Endothelial barrier dysfunction can result in the movement of both fluid and macromolecules into the interstitium and pulmonary air spaces. This can contribute to important morbidity and mortality (66). TNF can reduce capillary endothelial barrier function (67, 68).

## Regulation of AFC

In the normal lung, fluid and protein leakage is thought to occur primarily through small gaps between capillary ECs (2, 3). Since both capillary endothelial and AECs have TJ, fluid, and macromolecules that are filtered from the circulation into the alveolar interstitial space normally do not enter the alveoli (2).

The hydrophobic plasma membranes composed of phospholipids, act as a huge energy barrier for transporting ions (69–71). Yet, physiological processes assure for the continuous in- and outflow of ions, as such overcoming the plasma membrane barrier, which is impermeable to ions. Due to their biological complexity, interactions between cytokines and ion channels may be under-recognized (72). A group of plasma membrane proteins, including active transporters, generate and maintain ion concentration gradients for particular ions. These active transporters carry out this task by forming complexes with the ions they are translocating. The process of ion binding and unbinding for transport typically requires several milliseconds. As a result, ion translocation by active transporters is much slower than ion movement through ion channels, which can conduct thousands of ions across a membrane each millisecond. Active transporters effectively store energy in the form of ion concentration gradients, whereas the opening of ion channels rapidly dissipates this stored energy during relatively brief electrical signaling events.

Several types of active transporters have now been identified. Although the specific roles of these transporters differ, all must translocate ions against their electrochemical gradients (energetically “uphill”). Moving ions uphill requires the use of energy, and neuronal transporters fall into two classes based on their energy sources. Some transporters acquire energy directly from the hydrolysis of ATP and are called ATPase pumps. The most prominent example of an ATPase pump is the Na+/K+-ATPase pump, which is responsible for maintaining transmembrane (TM) concentration gradients for both Na^+^ and K^+^ (73). Another one is the Ca^2+^ pump, which provides one of the main mechanisms for removing Ca^2+^ from cells. The second class of active transporters does not use ATP directly as an energy source, but rather the electrochemical gradients of other ions. This type of transporter carries one or more ions up its electrochemical gradient, while simultaneously taking another ion, most often Na^+^, down its gradient. These transporters are usually called ion exchangers. An example of such a transporter is the Na^+^/Ca^2+^ exchanger, which shares with the Ca^2+^ pump the important task of keeping intracellular Ca^2+^ concentrations low. Other exchangers regulate both intracellular Cl^−^ concentration and pH by swapping intracellular Cl^−^ for another extracellular anion, bicarbonate, or the Na^+^/H^+^ exchanger that regulates intracellular pH, by regulating the concentration of H^+^. Although the electrochemical gradient of Na^+^ (or other counter ions) is the immediate source of energy for ion exchangers, these gradients ultimately depend on the hydrolysis of ATP by ATPase pumps, such as the Na^+^/K^+^ ATPase pump (74).

Alveolar fluid clearance is mainly regulated by Na^+^ uptake through the apically expressed ENaC and the basolaterally localized Na+/K+-ATPase in type II AECs (Figure 2, lower panel) (54). Dysfunction of these Na^+^ transporters during pulmonary inflammation can contribute to pulmonary edema (54). In this context, the movement of larger plasma proteins is restricted (2). The hydrostatic force for fluid filtration across the lung microcirculation is approximately equal to the hydrostatic pressure in the pulmonary capillaries, which is partly compensated by a protein osmotic pressure gradient (2). The net quantity of accumulated pulmonary edema is logically determined by the balance between the rate at which fluid is filtered into the lung (1) and the rate at which fluid is removed from the air spaces and lung interstitium (46). In mild-to-moderate lung injury, the capacity of the alveolar epithelium to transport salt and water is not only preserved but may also even be upregulated by stress hormones (Figure 3) (4). In severe lung injury, pulmonary fluid clearance can also be stimulated in lung injury by catecholamine-independent mechanisms (Figure 4) (4).

**Figure 6:**
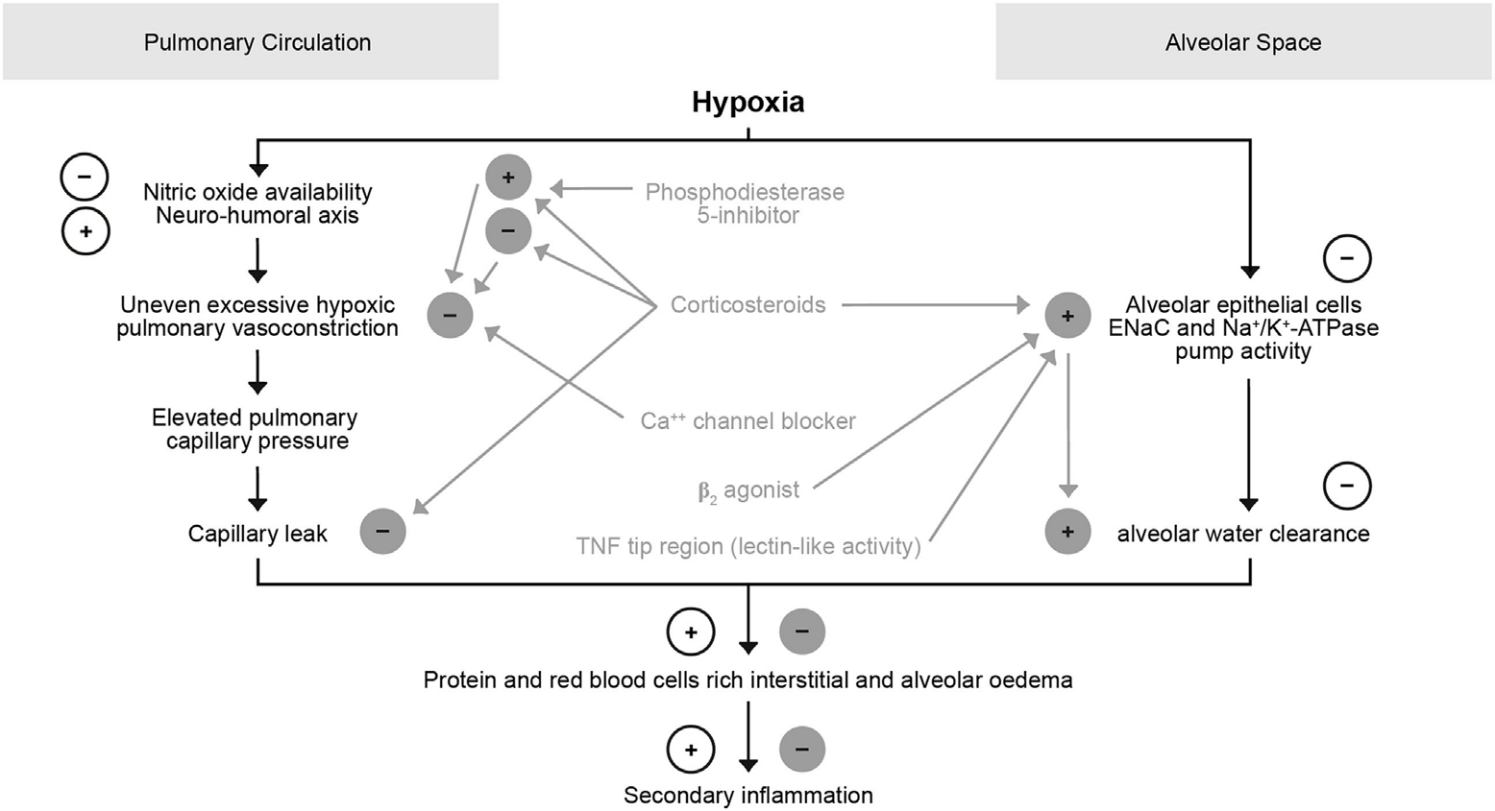
Role of hypoxia in the pulmonary circulation and alveolar space.

Moderate hypoxemia was shown to reduce AFC by 50%. This is caused by decreasing apical sodium uptake, at least partially through impaired trafficking of ENaC to the surface membrane (75–77). Hypoxia, moreover, inhibits the function of Na+/K+-ATPase in AECs, in part by triggering endocytosis through reactive oxygen species (ROS) and phosphorylation of the α1 subunit (78) (Figure 6). Restoration of normoxia rapidly reversed the depressant effects of hypoxemia in rats. Therefore, the simple administration of supplemental oxygen to patients with pulmonary edema may enhance the resolution of alveolar edema. As discussed more in detail in a contribution by Vadasz and Sznajder in this topic issue, hypercapnia can also impair AFC by the mechanisms of ubiquitination-mediated retrieval of ENaC from the plasma membrane, i.e., a post-translational modification of βENaC by regulating trafficking and stability, thereby modifying, and in this case reducing cell surface expression of the channel through βENaC ubiquitinylation in the alveolar epithelium (78–80). This mechanism seems of importance in ARDS as well in COPD. Hypercapnia and the associated acidosis have been shown to have anti-inflammatory effects, which might be advantages at sites of excessive inflammation, whereas on the other hand, ARDS and COPD studies showed that both patient groups had worse outcome when they were hypercapnic (78). In a randomized controlled trial Köhnlein, Windisch et al. showed that in severely sick, chronic hypercapnic COPD patients non-invasive ventilation, when targeted to reach noromocapnia (PaCO2 < 6.5 kPa/48.1 mmHg) or to improve hypercapnia by at least 20%, is associated with much better outcome (81). Survival was impressively improved, and also quality of life and lung function in terms of FEV1 improved. Possibly further effects exist such as sometimes improved cardiac output (82, 83), although interactions between ventilation and cardiac output are complex.

### Ion Channels and Pumps/Transporters and AFC

Ion channels are integral membrane proteins that form a pore to allow the passage of specific ions by passive diffusion (84). Most ion channels undergo conformational changes from closed to open states. Once open, ion channels allow the passage of thousands of ions (84). This distinguishes them from transporters and pumps, which can also transport ions, but only a few at a time (84). The opening and closing of channels can be controlled by various means, including voltage, the binding of ligands such as intracellular Ca^2+^ or extracellular neurotransmitters, and post-translational modifications such as phosphorylation (84).

Ion channels and pumps also play multiple important roles in cell homeostasis (84). Their function promotes passive, agonist-induced, or voltage-dependent flux of specific ions in and out of the cell (84, 85). The mchanisms of removing the infiltrated fluid from the alveoli is called AFC (84).

#### The ENaC in Type I and II Alveolar Epithelial Cells

Epithelial sodium channel, a member of the ENaC/degenerin (ENaC/DEG) family of ion channels, constitutes the rate-limiting entry step in Na^+^ reabsorption across epithelial in colon, kidney, and lungs (86). ENaC is inhibited by the drugs amiloride, benzamil, and triamterene, some of which are clinically used as potassium-sparing diuretics (87, 88). ENaC is a heteromultimeric protein (89) and is composed of at least four homologous subunits, α, β, γ, and δ (89–91) which are able to compose an ion channel (50, 92). A functional, pore-forming channel usually comprises one or two α subunits, together with a β - and a γ -subunit (89, 91, 93, 94). δ as a fourth unique subunit can form ion channels joining the β and γ subunits but exhibits biophysical and pharmacological features that are different compared to α ENaC channels (95). Investigations of the biological role of αENaC in the mouse lungs underlined the crucial role of this subunit in AFC (12). The β subunit is highly glycosylated and an important regulator of ENaC (4). In the lungs, ENaC is expressed not only in alveolar type II and type I cells (96), but also in capillary ECs (97).

Epithelial sodium channel was shown to exert a crucial role in pulmonary fluid reabsorption (46). Accordingly, ENaC is responsible for the maintenance of Na^+^ balance, extracellular fluid volume and blood pressure (98). ENaC activity is determined by the number of channels in the surface membrane *N*, which can change according to membrane insertion, degradation, or retrieval, as well as by the open probability time *Po* of individual channels (86, 99, 100). The basolaterally expressed, ouabain-inhibitable Na+/K+-ATPase then further drives the vectorial transport into the interstitium and, finally, into the lymphatic and blood vessels (73).

In order to maintain the correct composition and volume of alveolar lining fluid, Na^+^ transport through apically located ENaC in the alveolar epithelium is critical for gas exchange (92).

Epithelial sodium channel expression was shown to be decreased in transplanted lungs, both at the messenger RNA and protein level (8, 101).

#### Physiological ENaC Regulation

Epithelial sodium channel activity is important for fluid homeostasis and blood pressure control, but its regulation is complex and remains in many aspects incompletely understood (102) (Table 1). ENaC channels are also called highly selective cation (HSC) channels, and are presumed to be made up by the three ENaC subunits, α, β, and γ (103).

Epithelial sodium channel function can be affected by direct modulation of channel activity (92), subunit degradation, and membrane trafficking/recycling (104). cAMP indirectly increases ENaC activity, since it activates Cl^−^ uptake through CFTR (105). Intracellular as well as extracellular proteases, including prostasin and furin can affect the activity of the channel by modulating the Na^+^ self-inhibition (106, 107). Another important system that modulates ENaC activity is trafficking of the channels to the membrane, which involves a complex system of ubiquitination and binding to Nedd-4-2 (108). Na^+^ transport can also be regulated by gene expression (4). The two major hormonal modulators of pulmonary ENaC expression are catecholamines (50) and corticosteroids (109).

Many agents that increase Na+/K+-ATPase activity also increase ENaC activity (36). Negative ENaC regulators are activated purinergic P2Y receptors (110), NO (111, 112), Il-1β (113), hypoxia (46), and TGF-β (46).

#### ENaC Dysfunction

Dysfunction of the ENaC, which regulates salt and water homeostasis in epithelial, causes several human pathological conditions, including pulmonary edema (114). As ENaC regulates the airway surface liquid layer, its exaggerated activity might lead to airway dehydration, mucus stasis and bacterial overgrowth, as can be seen in cystic fibrosis and chronic bronchitis (115–117). ENaC hypo-activity, by contrast, can dramatically impair AFC, which is particularly important in conditions of pulmonary edema and correlates with mortality and morbidity in patients with ALI and ARDS (33).

The significant role of ENaC in inherited diseases associated with mutations in ENaC which increase or decrease channel activity regarding salt and water homeostasis has been well-documented (118). Mutations in the PPxY motif of β- and γ-subunits cause a severe form of hypertension, associated with ENaC in Liddle’s syndrome (OMIM: 177200) (119–123). A decrease in ENaC function can also cause a rare, life-threatening salt-wasting syndrome in pseudohypoaldosteronism type 1B (PHA1B) (OMIM: 264350) (124–127). This disease does not improve with age and patients are at risk from life-threatening, salt-losing crises, combined with severe hyperkalemia and dehydration throughout their entire lives (128, 129). Additionally, dysregulation of channel function and/or expression can lead to organ dysfunction and severe disease (84, 85, 130).

#### The Hybrid Acid-Sensing Ion Channel 1a (ASIC1a)/α-ENaC (NSC) Channels in Alveolar Type I and Type II Cells

Apart from ENaC, another apically expressed channel was recently shown to promote AFC. This hybrid channel is relatively non-selective for Na^+^ over K^+^, has a larger conductance, and shorter mean open and closed times (103, 131). In elegant assays, Trac et al. showed that the channel included ASIC1a as the mandatory counterpart to α-ENaC. These hybrid channels are, thus, composed of, at a minimum, one α-ENaC subunit and one or more ASIC1a subunits. The biological significance is great, as the regulation of these NSC channels is dramatically different from ENaC. Thus, treatments to reduce alveolar flooding based on the known properties of ENaC (HSC) could be suboptimal because ASIC1a/α-ENaC-channels are regulated differently (see Table 3). Indeed, NSC channels are less sensitive to inhibition by amiloride than ENaC HSC channels.

As the proton-gated ASIC1a plays a role in the formation of channels, its properties determine the pharmacological ASIC1a/α-ENaC-channels (NSC) modulation. The MitTx agonist, derived from Texas coral snake toxin, strongly activates ASIC1a/α-ENaC-channels (NSC) (Table 3).

#### Why Do Alveolar Epithelial Cells in the Lungs have Several Types of Channels That Mediate Na^+^ Uptake?

As shown, an important functional role of non-selective cation (NSC) channels, which consist of ASIC1a and of ENaC-α subunits (281), is Na^+^ uptake by AT2 cells in the lung (103). By contrast, other sodium-transporting epithelial tissues such as the distal nephron of the kidney and the colon were not reported to have these functional NSC channels, and mainly transport Na^+^ through ENaC. In the lungs, the alveolar fluid layer must be very tightly controlled. Therefore, it may be important to have alternative ion transport pathways that respond differently to physiological stimuli, such as to acidification, which accompanies ALI and which activates NSC channels (282). An alternative hypothesis is that NSC channels provide a stable driving force for cation and anion movement across the alveolar epithelium. Indeed, NSC channels contribute to the apical membrane potential, causing the membrane potential to be close to zero. This will ensure that there is a driving force for the unidirectional movement of anions, through CFTR and for movement of Na^+^ through classical ENaC and NSC into cells. This is necessary because of the requirement to move salt, i.e., anions plus cations. Other epithelia tend to have counter-ion pathways for cations that obviate the need to maintain a strong potential driving force.

In an evolutionary context, the lung has been the most recent organ to adapt to a terrestrial environment. Typical for evolutionary processes is the modification of existing mechanisms to produce a different evolutionary outcome, in this case, the formation of a new channel type out of parts from two pre-existing channels of the same channel family. Of further evolutionary interest is that the activity of both HSC channels HSC (ENaC) and NSC channels is increased by a peptide mimicking the lectin-like region of TNF, which binds to ENaC-α, as shown below and in Czikora et al. (9), in this issue (9).

### The Na+/K+-ATPase

Apart from apical ENaC and, potentially NSC, the basolaterally expressed Na+/K+-ATPase, a.k.a. the sodium-potassium pump is also a crucial driver of AFC (73, 78). Na+/K+-ATPase activity regulation also involves complex patterns, including modulation of the trafficking of the protein to the membrane (73). The Na+/K+-ATPase is a ubiquitous enzyme consisting of α and β subunits and a less well-characterized regulatory FXYD subunit. The Na+/K+-ATPase is responsible for the generation and preservation of the Na^+^ and K^+^ gradients across the cell membrane by transporting 3 Na^+^ out and 2 K^+^ into the cell (283).

Changes in intracellular Na^+^ concentration and hormones, such as mineralocorticoids, glucocorticoids and thyroid hormones as well as adrenoceptor stimulants modulate Na+/K+-ATPase activity (284). Like ENaC, increase of Na+/K+-ATPase expression is considered central to enhance transepithelial Na^+^ transport (4). In addition, thyroid, mineralocorticoid and glucocorticoid hormones modulate Na+/K+-ATPase expression (4). Likewise, β adrenoceptor activation upregulates Na+/K+-ATPase expression in AECs (50).

The Na+/K+-ATPase contains one principal catalytic subunit, designated α and one sugar-rich auxiliary subunit, designated β. There is also a regulatory subunit FXYD subunit, which was recently shown to play an important role in regulation of lung inflammation (285). The α-subunit carries the catalytic function of the enzyme, and this is reflected in its possession of several binding and functional domains (283). The α subunit (4) transports Na^+^ out of the cell, providing the driving force for Na^+^ reabsorption (286). It is clear that an essential role for β subunit lies in the delivery and the appropriate insertion of the α subunit in the membrane (287). In recent years, a variety of studies have suggested that the β subunit may be more intimately involved in the mechanism of active transport (287–290).

FXYD5 or Dysadherin or RIC is a pro-inflammatory type I membrane protein, which belongs to seven members of the FXYD family named by their shared TM amino acid motif. FXYD5 is an established tissue-specific modulatory subunit of Na+/K+-ATPase, expressed in a variety of epithelial cells. Recent work shows a role for FXYD5 as a key mediator of the inflammatory response during ALI (285). It impairs adherens junctions by downregulating the markers zona occludins-1 (ZO-1) and occludin and redistributing beta catenin (291). It is required for the secretion of NF-κB, e.g., upon lipopolysaccharide (LPS), and inflammatory mediators, including TNF and interferon-α (IFN-α) and C-C chemokine ligand-2 (CCL2) from AECs that activate alveolar macrophages, amplify lung injury by orchestrating an overly exuberant inflammatory response, and recruit monocytes into the alveolar compartment, or in bronchoalveolar lavage fluid (285). The presence of FXYD5 is an important component for NF-κB activation pathway as shown in AECs induced by LPS, TNF, or interferon-α, as its silencing prevented IκB-α phosphorylation and reduced cytokine secretion in response to these stimuli. Probably FXYD5 increases CCL2 transcription by inducing Akt-dependent activation of NF-κB signaling. Binding of IFN-α activated phosphoinositide 3-kinase (PI3K) *via* STAT5, which in turn activates NF-κB. Activation of PI3K seems downstream of TLR4 and TNFR1. Possibly, FXYD5 modulates NF-κB signaling by regulating the location of TNF receptor 1, by modulation associations with other proteins and their location and mobility in the membrane (285). It is of interest that FXYD5 regulates inflammation, activates NF-κB dependent cytokine secretion and infiltration of immune cells to the alveolar spaces as well as alveolar barrier tightness, and is closely linked to one key ion transport channel.

### The Cystic Fibrosis Transmembrane Conductance Regulator

Cystic fibrosis transmembrane conductance regulator is a cAMP-regulated and post-translationally modified chloride channel of 1,480 amino acids, which is mainly expressed in epithelial cells. The non-glycosylated form of CFTR has a molecular weight of 127 kDa, with 160 kDa for the glycosylated form. CFTR can either take up or release Cl^−^ ions from the AT1 and AT2 cells. Apical to basolateral chloride transport may be important because the maximal rate of sodium and water transport from the airspaces appears to be limited by the concomitant chloride transport (115–117). An important part of transepithelial chloride transport occurs through the paracellular route in the alveolar epithelium. The selectivity and magnitude of paracellular ion conductance may influence net transport capacity. Upon increasing Cl^−^ influx, CFTR will activate ENaC-mediated Na^+^ uptake, as such activating AFC, but the channel will inhibit AFC upon increasing Cl^−^ efflux. Increased cAMP generation will open CFTR in the apical membrane of AT1 and AT2 cells for Cl^−^ uptake, as such increasing Na^+^ uptake and AFC. Therefore, factors that can activate cAMP-mediated Cl^−^ uptake by CFTR, such as β2 agonists, have been investigated as potential therapeutic candidates for pulmonary edema (105). Cystic fibrosis, a disease characterized by impaired airway dehydration, is caused by a loss of function of CFTR, accompanied by an excessive activity of ENaC. A peptide mimetic of SPLUNC, i.e., SPX-101, was shown to promote internalization of the three ENaC subunits and to restore mucus transport in a mouse and a sheep model of CF (292).

### The Transient Receptor Potential Vanilloid 4 (TRPV4) Channel

Transient receptor potential vanilloid 4 is a TM cation channel and a vanilloid-type member of the transient receptor potential (TRP) protein superfamily (293). TRPV4 is ubiquitously expressed in many cell types in the respiratory system (294). It is part of an integrated system, consisting of ion channels and membrane pumps, which tightly regulates intracellular calcium levels in a spatiotemporal manner (295). TRPV4 counts 871 amino acids and contains six TM domains, an ion pore located between TM5 and 6, an NH2 terminal intracellular sequence with several ankyrin-type repeats, and a COOH-terminal intracellular tail (296, 297). Both the NH2 and COOH termini interact with signal kinases, other molecules (e.g., NO), and scaffolding proteins (298). The intracellular tails contain several activity-modifying phosphorylation sites (294). In the setting of pulmonary inflammation, TRPV4 has been found to be highly expressed and upregulated in airway smooth muscle, vascular ECs, AECs, as well as in immune cells, such as macrophages and neutrophils (298–303).

#### The Role of TRPV4 in Pulmonary Edema

Transient receptor potential vanilloid 4 mediates cellular responses to both physical (such as osmotic, mechanical, and heat) as well as chemical stimuli (304). It is also involved in lung diseases associated with parenchymal stretch and inflammation or infection (254, 294). Target diseases include cough, asthma, cancer, and pulmonary edema associated with ARDS (253, 294, 305–310).

These studies support a role for TRPV4 in a broad spectrum of lung and airway functions and disease processes. TRPV4 also has been implicated as a key regulator of lung endothelial barrier integrity, specifically, the integrity of the lung alveolar–capillary endothelium, which is most relevant to alveolar edema generation in ALI (311). TRPV4 activation increases vascular permeability, thus promoting protein and fluid leak (254).

Several studies have shown that TRPV4 can regulate generation of inflammatory cytokines that play key roles in orchestrating lung tissue homeostasis and inflammatory lung disease (301, 307, 309, 310, 312–314). Therefore, TRPV4 could be considered a potential target for lung disease pathogenesis, including to alveolar–capillary barrier function (300). TRPV4 has been proposed as a candidate target for the management of ALI that develops as a consequence of aspiration of gastric contents, or acute chlorine gas exposure (254). Protection from the ALI response to intratracheal HCl and a key role *in vivo* of polymorphonuclear neutrophil TRPV4 (294) was noted in mice that lack TRPV4 (TRPV4 KO), or in mice that were treated with three different small molecule inhibitors of TRPV4 (253, 301, 307, 309, 312, 313, 315).

However, in view of its ubiquitous expression, and the multitude of functions attributed to the channel, including its role in pulmonary vasomotor control, endothelial barrier tightness, inflammatory response and systemic blood pressure regulation, TRPV4 blockade may represent a double-edged sword. Therapeutic benefits of TRPV4 inhibition have, therefore, to be carefully weighed against potential adverse effects (254).

Transient receptor potential vanilloid 4 activation and its downstream signaling pathways differ in response to varying stimuli, cell types, and contexts (294). For instance in asthma, TRPV4 mediates hypotonicity-induced airway hyperresponsiveness, but not release of Th2 cytokines (312, 316). In CF, TRPV4 appears to play paradoxical roles in CBF/mucociliary clearance and epithelial cell pro-inflammatory chemokine (IL-8/KC) secretion (317, 318). Depending on the underlying etiology, TRPV4 may play different roles in ARDS (307, 310, 314, 319). Also, in pulmonary fibrosis, TRPV4 has been shown to mediate the mechano-sensing that drives myofibroblast differentiation and experimental lung fibrosis in mice (308).

#### TRPV4 and Macrophage Function in Lung Injury

Alveolar macrophages are known to be effector cells in bacterial and particle clearance but also in any injury and repair process (320). Since intracellular Ca^2+^ is known to be required for the phagocytic process, and because TRPV4 plays a role in force-dependent cytoskeletal changes in other systems/cell types, the role of TRPV4 in macrophage phagocytosis was extensively studied by Scheraga and colleagues (213, 253, 307, 315, 321–323). The process of phagocytosis in macrophages requires integration of signals from macrophage surface receptors, pathogens, and the extracellular matrix (324–326). However, the effects of matrix stiffness on the macrophage phenotypic response or its signal transduction pathways have yet to be fully elucidated (294). TRPV4 mediates LPS-stimulated macrophage phagocytosis of both opsonized particles [immunoglobulin G (IgG)-coated latex beads] and non-opsonized particles (Escherichia coli) *in vitro* (294). Inhibition of TRPV4 by siRNA or pharmacologic inhibitors completely abrogated both the LPS effect and the matrix stiffness effect on phagocytosis (294). These data indicate that both the LPS and stiffness effect on macrophage phagocytosis are TRPV4 dependent (310). Concordant with their *in vitro* data, also LPS-induced alveolar macrophage phagocytosis was proposed to be TRPV4 dependent (294).

Collectively, obtained data demonstrate that TRPV4 responds to extracellular matrix stiffness, thereby altering the LPS signal to mediate macrophage phagocytosis and cytokine production (310). Furthermore, TRPV4 regulates a feed-forward mechanism of phagocytosis in activated lung tissue macrophages when they interact with stiffened infection/injury-associated lung matrix. This concept is further supported by the observation that surfactant protein B-deficient mice have altered alveolar macrophage shape and function in association with increased alveolar surface tension (327).

### Other Ion Channels

Recent research has given much more detail to a number of further ion channels and their interactions, such as Cl^−^ regulators in the paracellular TJ area including claudin-4 and -18 implicated in epithelial ion and fluid transport and ARDS regulation in specific infectious, inflammatory, or other stimulatory situations. The reader is referred to further reviews as that of Brune et al (11). and Weidenfeld and Kübler (5). The transient receptor potential channel 6 (TRPC6), a Ca^2+^-permeable non-selective cation channel, widely expressed in the lungs, was proposed to be a key regulator of acute hypoxic pulmonary vasoconstriction and was demonstrated to be implicated in pulmonary hypertension. TRPC6 is also involved in pulmonary vascular permeability and lung edema formation during LPS- or ischemia/reperfusion-induced ALI as discussed in this topic issue (328).

## Cytokine-Ion Channel Interaction

Cytokines, which are organized in a cytokine network, play a major role in maintaining lymphocyte and leukocyte homeostasis under both steady-state and inflammatory conditions (329). Regulatory cytokines have to function in combination with other environmental signals to properly modulate the function and the extent of lymphocyte and leukocyte activation (329). Increased generation of pro-inflammatory cytokines represents a first-line defense mechanism against bacterial infections of the lung (102). Dysregulation of cytokine generation leads to alterations in cell–cell interactions (330). Cytokines, such as TNF, IL-1, IL-6 activate host defense by promoting the production of a wide spectrum of other cytokines and chemokines, including GM-CSF, G-CSF and IL-8 in inflammatory processes (331, 332). They moreover mediate the increase of surface adhesion molecule expression through activation of leukocytes and ECs (38). As such, cytokines can contribute to the pathogenesis and development of pulmonary edema (37, 99, 333–338). During the acute phases of ARDS, higher levels of TNF were detected in the BALF from patients with early-stage ARDS (39).

### The Dichotomous Yin and Yang Effects of TNF in Pulmonary Edema

Tumor necrosis factor is a homotrimeric 51 kDa protein, binding to two types of membrane receptors: TNF receptor 1, which signals either apoptosis, necroptosis or inflammation; and TNF receptor 2, which is mainly implicated in inflammation and which is devoid of a death domain (239, 339, 340). TNF is one of the central cytokines in inflammation and moreover modulates ion channel activity (341–344). An intriguing feature of the ligands of the TNF and TNFR family is that when certain members are shed, they inhibit the function of the ligand-receptor complex and act as inhibitors (345). A central regulatory process may, therefore, be the proteolytic release of soluble bioactive oligomers from membrane-bound forms, e.g., for TNF by the protease TACE. The existence of TM forms of most of the TNF-superfamily ligands indicates that they are meant to act locally. Only under non-physiological conditions, when these ligands are released, they may prove to be harmful (345) or beneficial, as is the case of immune defense to bacterial infection (346). As a consequence, long-term treatment with TNF neutralizing substances can cause increased sensitivity to tuberculosis (346).

**Figure 7:**
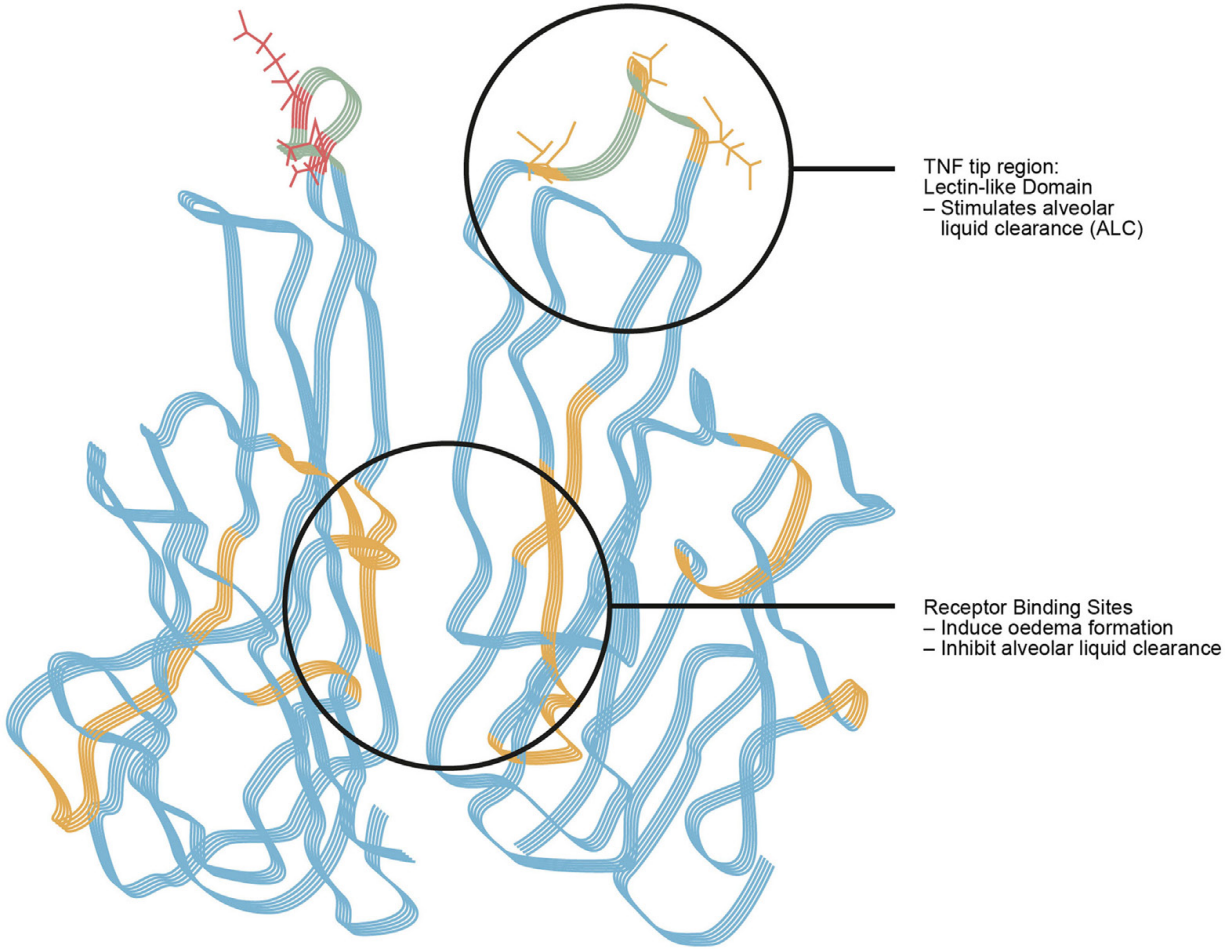
Tumor necrosis factor. Tumor necrosis factor (TNF) as a “moonlighting” or dual role, or dichotomal yin-yang cytokine. The TNF receptor 1 binding sites within the TNF homotrimer mediate edema formation and blunt edema reabsorption. The lectin-like domain of the same cytokine activates epithelial sodium channel function and as such promotes alveolar fluid clearance and acts on endothelial cell barrier tightness (360).

Tumor necrosis factor contributes to the pathogenesis and development of pulmonary edema (38), but, paradoxically, also plays an important role in edema reabsorption (347–350). It was assumed for a long time that cytokines exert their activities solely upon activating their respective receptors, but in the case of TNF, this is not true, which broadens this concept (38). TNF was shown to exert a lytic, i.e., killing effect on certain bloodstream stages of African trypanosomes, by means of a lectin-like interaction with trimannoses and *N,N*’-diacetylchitobiose oligosaccharide residues in the variant surface glycoprotein on the surface of the parasites (344). Later investigations could demonstrate that this lectin-like activity can be attributed to a special 17 amino acid long domain, named the lectin-like domain of TNF in the molecule’s tip region (351, 352) (Figure 7). This special region is spatially distinct from its receptor binding sites (353) and is not present in lymphotoxin, which has a highly similar tertiary structure as TNF. Comparative sequence analysis of TNF and LT allowed for the identification of the lectin-like domain of TNF (353).

For experimental purposes to mimic the TNF lectin-like domain, the amino acid sequence-identic synthetic 17 amino acid peptide which has shown to biologically mimic the lectin-like tip domain of TNF (353–355), as described above, has been used in a variety of experimental researches. It, moreover, gave rise to a therapeutic candidate that was recently evaluated in clinical trials (a.k.a AP301 and Solnatide) (356–358).

There are conflicting data about the critical involvement of TNF in the regulation of AFC (359). *In situ* and *in vivo* investigations conducted by Braun et al. in flooded rat lungs demonstrated a dual role for TNF in pulmonary edema (37, 38). This is possibly due to the opposite effects of, on the one hand, the classical TNF receptor 1 binding sites and, on the other hand, the lectin-like domain of TNF on pulmonary fluid reabsorption (37). In fact, the TNF tip region with its lectin-like activity is spatially distinct from the cytokine’s receptor binding sites and causes an increase of alveolar fluid reabsorption, which is completely independent of the TNF receptors type 1 and 2, and further increases the cell–cell barrier tightness as shown in the alveolar EC barrier (Figure 7) (38, 99).

As discussed more in detail in this issue (361), in murine models of ventilator-induced ALI, TNF receptor 2 can have protective effects, whereas TNF receptor 1 is deleterious, thus adding another level of complexity to the role of TNF in edema (362). As such, the complex between soluble TNF receptor 1 and TNF can stimulate fluid reabsorption. TNF causes receptor-mediated edema formation in part by decreasing the expression of ENaC mRNA in AECs *in vitro* (135) leading to decreased amiloride-sensitive sodium uptake (135). Moreover, TNF receptor 1 signaling initiates the process of neutrophil migration (363) which can also contribute to the formation of pulmonary edema. It is also involved in orchestrating mechanisms, such as complement activation, cytokine regulation, chemokine production, and activation of adhesion molecules as well as their respective adhesion molecule receptors (364).

A TNF-dependent and amiloride-sensitive increase in AFC occurs in a rat model of *Pseudomonas aeruginosa* pneumonia (365). Other studies have shown in rats that intestinal ischemia–reperfusion leads to stimulation of AFC. This stimulation is at least in part mediated by a TNF-dependent mechanism which is independent of catecholamine release, because propranolol did not influence the AFC, and there was no observed cAMP stimulation (366). This indicates a protective effect of TNF-dependent stimulation of AFC in the early phase of injury (366).

Fukuda et al. could show that in ventilated rats TNF increased AFC by about 67% (136). This increase was inhibited by amiloride, but not by propranolol, indicating the mechanism is catecholamine-independent. A triple TNF mutant, in which three crucial residues for the lectin-like activity were mutated to alanines, did not show any increase in AFC. The effect of TNF occurred within 30 s from the onset of perfusion in A549 cells and within 1 h in the distal airspaces of the rat. This shows that the primary mechanism does not depend on a transcriptional effect of TNF. This indicates that TNF increased AFC most probably by an amiloride-sensitive mode of action, independent of any TNF receptor binding and mediated through the lectin-like region.

These antagonistic functions of the same molecule on pulmonary edema refer to the complex biology of the TNF molecule (361). Indeed the TNF receptor 1 binding sites of TNF inhibit, whereas its lectin-like domain activates edema reabsorption (Figure 7) (37), and, as described above, tightens intercellular epithelial and endothelial barrier function (8, 9).

### The Impact of TNF on Pulmonary Edema Generation by TNF Receptor-Mediated Effects

Tumor necrosis factor is mainly known for its receptor-mediated pro-inflammatory functions in the systemic inflammatory response and the induction of apoptosis on a cellular level (339, 367). Both of these activities of TNF are implicated in the pathogenesis of pulmonary edema, which is often associated with ALI (37).

Tumor necrosis factor promotes pulmonary dysfunction through edema formation and inhibition of edema reabsorption by several procedures (37), for instance:

- TNFR-dependent upregulation of chemokine production (338, 363) and adhesion molecule expression (333, 334, 368), which leads to neutrophil attraction and sequestration.
- Decrease in barrier function in human pulmonary artery ECs and rearrangement of microtubules (67).
- Induction of reactive oxygen intermediates (336).
- Down-regulation of ENaC expression in alveolar type 2 cells (135)

#### TNF Inhibits Transcription of All Three ENaC Subunits

Seminal studies conducted by Dagenais et al. clearly demonstrated the involvement of TNF in modulation of Na^+^ absorption in cultured AECs is investigated. The results show that TNF decreased the expression of the α-, β-, and γ-subunits of ENaC mRNA after 24-h treatment and reduced to 50% the amount of ENaC-α protein in these cells (135). There was no impact, however, on α1 and β1 Na+/K+-ATPase mRNA expression (135). Amiloride-sensitive currents and ouabain-sensitive Rb^+^ uptake were reduced. A strong correlation was found at different TNF concentrations between the decrease of amiloride-sensitive current and ENaC-α mRNA expression (135). All these data show that TNF has a profound effect on the capacity of AECs to transport Na^+^ (135). In another study performed by Yamagata et al., mRNA expression of all three ENaC subunits in whole lung tissue was inhibited by TNF (359). TNF also inhibited ENaC function, as indicated by the reduction of amiloride-sensitive current (359). These data suggest that TNF may affect the pathophysiology of ALI and pulmonary edema through the inhibition of AFC and sodium transport (359).

#### TNF Increases Permeability of the Epithelial–Endothelial Barrier

The activation of TNF receptor 1 by TNF modulates the integrity of the alveolar barrier, in addition to its direct effects on ion channels and pumps of the alveolar epithelium. TNF increases the endothelial expression of chemo-attractants and adhesion molecules including IL-8 (formerly called neutrophil chemotactic factor), the IL-8- receptor 2, the intercellular adhesion molecule-1 (ICAM-1), platelet endothelial cell adhesion molecule-1 (PECAM-1), and vascular adhesion molecule-1, thus promoting excessive recruitment of mononuclear phagocytes and neutrophils during lung inflammation (71, 369–371).

Tumor necrosis factor is released in acute inflammatory lung syndromes linked to the extensive vascular dysfunction associated with increased permeability and EC apoptosis (372). The critical importance of the pulmonary vascular barrier function is shown by the balance between competing EC contractile forces, which generate centripetal tension, and adhesive cell–cell and cell matrix tethering forces, which regulate cell shape. Both competing forces in this model are intimately linked through the endothelial cytoskeleton, a complex network of actin microfilaments, microtubules, and intermediate filaments, which combine to regulate shape change and transduce signals within and between ECs (66).

Tumor necrosis factor can activate ECs, cause acute pulmonary vascular endothelial (VE) injury or even EC death and increase pulmonary vascular permeability *in vivo* as well as *in vitro* (39, 67, 373). Also, TNF increases the permeability of EC monolayers to macromolecules and lower molecular weight solutes by involving pertussis toxin-sensitive regulatory G protein (374). Furthermore, it is reported that TNF can increase the permeability of lung EC monolayers and that fibronectin can blunt this effect (375). In addition, TNF-induced increase in endothelial permeability involves the loss of fibronectin and remodeling of the extracellular matrix (376). Moreover, it has also been shown that TNF can increase capillary permeability causing transcapillary filtration *in vivo* (377).

#### TNF Increases ROS Generation

In addition to the above-mentioned mechanisms, TNF can induce pulmonary edema indirectly through increasing ROS (336). ROS have been shown to be able to disrupt the pulmonary endothelial barrier (336) and to decrease Na^+^ channel activity (378).

### Identification of the Alveolar Liquid Clearance-Promoting Effects of TNF

#### Lung Transplantation and Primary Graft Dysfunction (PGD)/Ischemia–Reperfusion Injury

The receptor-independent lectin-like domain of murine TNF has a potential physiological role in the resolution of alveolar edema in an *in situ* mouse lung model and an *ex vivo* rat lung model (99). The lectin-like domain of TNF can activate amiloride-sensitive sodium uptake in type II AECs (99, 100). Therefore this TNF domain is a potential therapeutic candidate (360).

As there is no specific treatment for ischemia–reperfusion-mediated lung injury, which is accompanied by a disrupted capillary barrier integrity and an impeded AFC, the capacity of the TNF tip peptide to improve lung function after unilateral orthotopic lung iso-transplantation was tested *in vivo* in adult rats (8).

The unilateral rat transplant study showed that a highly severe lung injury with blood gas parameters qualifying for severe ARDS could be virtually prevented by the activation of the TNF lectin-like region. Furthermore, a significant reduction in polymorphonuclear neutrophilic leukocytes (PMN) infiltration in the bronchoalveolar lavage fluid was observed. The TNF tip peptide reduced ROS generation in the transplanted rat lungs *in vivo* and diminished ROS generation in pulmonary artery ECs *in vitro* under hypoxia and reoxygenation (8). ROS, the generation of which is increased during ischemia–reperfusion ALI (379–381), have been shown to be able both to disrupt pulmonary endothelial barrier integrity (378) and to inhibit ENaC activity (382).

Moreover, the effect of the lectin-like domain of TNF likely has physiologic relevance during inflammation and infection (8). As the soluble TNF receptors are cleaved by the same enzyme that generates soluble TNF, i.e., TACE (383), complexes between soluble TNF receptors and TNF can form (8). Soluble TNF receptors do not inhibit the activity of the lectin-like domain of TNF and complexes between these receptors and TNF are even able to stimulate AFC in *in situ* flooded rat lungs (37, 99, 353). At the same time, unfavorable actions of TNF on edema reabsorption and formation that are mediated by TNF receptor 1 activation are being blocked by the soluble receptors (37). Therefore, the favorable actions of the lectin-like domain of TNF might occur in conditions where both TNF and its soluble receptors are being generated (8).

A recent pilot study of 20 patients on treatment of PGD by twice daily nebulized 125 mg inhalation of the TNF tip peptide (AP301, solnatide) randomized 1:1 showed an improved gas exchange (mean and SD, daily measured up to 72 h, PaO2/FiO2 365.6 ± 90.4 versus 335.2 ± 42.3 mm Hg; *p* = 0.049) and clearly less time intubated (2 ± 0.82 versus 3.7 ± 1.95 days, p = 0.02) in the verum group, which also seems clinically relevant (357).

In summary, the lectin-like activity of TNF, and thus, the TNF tip peptide significantly improves lung function after lung transplantation in the rat. Pilot studies confirm a relevant effect in clinical treatment (8, 357). The experimental model showed a reduced alveolar neutrophil content and less ROS generation. It exerts a favorable effect on organ function in terms of gas exchange (8). It was furthermore shown that the apically expressed ENaC was found to be decreased at the messenger ribonucleic acid and the protein level in transplanted lungs, suggesting that ENaC, rather than the basolaterally expressed Na+/K+-ATPase, is important in the abnormal AFC (101). These studies reinforce the idea that the TNF tip peptide acts as an agent with potential therapeutic traits against the ischemia–reperfusion injury associated with lung transplantation.

#### The Lectin-Like Region of TNF Ameliorates High-Altitude Pulmonary Edema (HAPE) in Rats

About 100 million people live at altitudes greater than 2,500 m, about 15 million above 3,000 m, and some above 5,000 m (384). Most of these individuals have developed the ability to live and reproduce at elevation as high as 5,000 m, but in some cases, develop chronic medical problems due to their high-altitude residence. At 5,500 m barometric pressure is about only half of the one at sea level. Furthermore, many lowlanders venture to high altitude for work and recreation. The prevalence of HAPE depends on an individual’s susceptibility, the rate of ascent, the final altitude, but also on heavy and prolonged exercise, and is higher in males (385). Although the mechanism underlying HAPE remains incompletely understood, it appears that the elevated pulmonary artery pressure plays a pivotal role in the process. Multiple studies demonstrated that susceptible individuals have abnormally high pulmonary artery pressure in response to hypoxic breathing, during normoxic and hypoxic exercise, and on high altitude before the onset of edema. Increased sympathetic tone, and alteration in vasoactive mediators such as endothelin-1, NO produced by pulmonary ECs, may also lead to stronger hypoxic pulmonary vasoconstriction (384). In autopsies, a red cell rich proteinaceous alveolar exudate with hyaline membrane is characteristic. In all autopsies, areas of pneumonitis with neutrophil accumulation but no evidence of bacterial accumulation have been observed. The estimated death rate of altitude illness is about 7.7/100,000 trekkers, with increasing mortality during the last decade (386). Treatment of HAPE consists, if ever possible, in descent from altitude, rest, oxygen supplementation, and administration of drugs like corticosteroids and furosemide.

Prophylactic inhalation of salmeterol, an inhalative β2-adrenergic receptor (β2AR) agonist, decreased the incidence of HAPE by more than 50% (387). The most pertinent explanation was that salmeterol would enhance the clearance of alveolar fluid since β2-adrenergic agonists upregulate AFC by stimulating transepithelial sodium transport. This hypothesis is supported by the fact that the level of sodium transport in the respiratory epithelium is lower in patients prone to HAPE. However, the study results cannot exclude the possibility that the β2 agonist could have modulated vascular permeability or the hemodynamic response associated with hypoxemia and HAPE (4).

In an experimental rat model simulating HAPE by hypobaric and hypoxic conditions equivalent to an altitude of 4,500 m with exhaustive treadmill exercise of 15 m per minute for 24 h, then for an equivalent of altitude of 6,000 m for further 48 h, the TNF tip peptide reduced pulmonary edema and increased expression of the epithelial TJ protein occludin, as compared to high-altitude controls. Compared to untreated high-altitude control animals, TNF tip peptide significantly lowered levels of the inflammatory cytokines TNF, IL-1β, IL-6 and the chemokine IL-8 in bronchoalveolar lavage. TNF tip peptide-treated animals experienced less pulmonary edema, as compared to dexamethasone-treated animals, and was more effective than its comparators in reduction of bronchoalveolar lavage protein content and inflammatory parameters (7).

#### Identification of the Mechanism of ENaC Activation by the Lectin-Like Region of TNF

It has been shown that the lectin-like domain of TNF can activate ENaC (353) and increases sodium uptake capacity in type II AEC (38). Intriguingly, the TNF tip peptide was shown to directly bind to the α subunit of ENaC (54, 102) in a two-hit manner, first interacting with the glycosylated extracellular loop of the subunit and subsequently in the TM 2 domain, where the actual activation of the channel occurs (54, 102, 114). The former interaction was proposed to increase the expression of ENaC at the surface membrane in the presence of bacterial toxins, whereas the latter increases the channel’s open probability time (102). Indeed, the binding of ENaC to the lectin-like domain of TNF or to the TNF tip peptide stabilizes the channel’s complex formation with myristoylated alanine-rich C kinase substrate and with phosphatidylinositol 4,5-bisphosphate, both of which are important for the open conformation of the channel (388), in the presence of the pneumococcal pore-forming toxin pneumolysin (PLY), an important mediator of permeability edema in pneumococcal pneumonia (54). Knock-in mice expressing a TNF mutant lacking a functional lectin-like domain was shown to be more prone to develop capillary leak and permeability edema than their wild-type counterparts after instillation of a low dose of PLY, which did not induce significant barrier dysfunction in control mice (54). In short, these results demonstrate a novel TNF-mediated mechanism of direct ENaC activation and indicate a physiological role for the lectin-like domain of TNF in the resolution of alveolar edema during inflammation (54).

#### The Lectin-Like Region of TNF Increases Activity of Na+/K+-ATPase

Vadasz et al. investigated the impact of the TNF tip peptide on fluid balance in experimental lung injury. Alveolar–capillary permeability and fluid clearance were assessed in adult male rabbits. Aerosolized TNF tip peptide improved ALC by both reducing vascular permeability and by enhancing the absorption of excess alveolar fluid in experimental lung injury. TNF tip peptide increased Na+/K+-ATPase activity by promoting its exocytosis to the AEC surface and increased amiloride-sensitive sodium uptake, which increased the active Na^+^ transport 2.2-fold and consecutively the AFC (196). Together with its previously discussed effects on ENaC, these data suggest a role for the TNF tip peptide as a potential therapeutic agent in pulmonary edema (196), since the two main mediators of Na^+^ transport are both activated by the TNF tip peptide. It should be noted that the primary target is likely ENaC and that the activation of Na+/K+-ATPase could be through the indirect increase in intracellular Na^+^ upon prior stimulation of ENaC (8). Moreover, the TNF tip peptide was recently also shown to increase the activity of NSC channels (9).

#### The Lectin-Like Region of TNF Restores ENaC Function in PHA1B Mutants

The lectin-like domain of human TNF activates the ENaC in various cell- and animal-based studies. The synthetically produced cyclic peptides Solnatide (a.k.a. tip peptide or AP301) and its congener AP318 possess molecular structures that mimic the TNF tip region. AP318-mediated ENaC activation was shown to rescue loss of function in a phenotype of ENaC carrying mutations and restored the amiloride-sensitive Na^+^ current to physiological levels or even higher (118). This implies that the TNF tip domain can activate ENaC by a mechanism which remains intact even in the presence of various mutations occurring in different subunits, because binding to the putative binding site in the TM 2 domain of the glycosylated α subunit apparently remains basically unaffected in all tested point mutations or was compensated in frame shift mutations *via* a moderate activation of αβ- and βγ-ENaC, respectively (389). Apart from the mechanism responsible for loss of the ENaC performance in the studied ENaC mutations, the synthetic TIP and AP318 peptides could restore ENaC function up to or even higher than current levels of wild-type ENaC (118). As therapy of PHA1B is only symptomatic so far, these TNF tip peptides, which directly target ENaC, are promising candidates for the treatment of the channelopathy-caused disease PHA1B (118).

#### Clinical Trials on the Effect of the Lectin-Like Region of TNF

In a recent phase 2a clinical trial with ALI, patients received inhalable TNF tip peptide in the ventilator twice daily over a 7-day period. There was no significant improvement in lung liquid clearance over all patients, as assessed by the PiCCO method. However, there was a significant increase in extravascular lung water removal in those patients with a sequential organ failure assessment score higher than or equal to 11, representing more than 50% of the subjects in this trial (358). One hypothesis for this observation is that patients in this group, apart from suffering from impaired AFC capacity, might also suffer from more severe capillary barrier dysfunction. The TNF tip peptide was recently shown to not only improve AFC (54, 102), but also capillary barrier function (97) in the presence of bacterial toxins.

As mentioned before, in a randomized pilot study performed with 20 patients on the treatment of established PGD after lung transplantation by twice daily inhalation of the TNF tip peptide (AP301, solnatide) versus placebo, the TNF tip peptide improved gas exchange and clearly reduced the intubation—and thus mechanical ventilation—time in a probably clinically relevant manner (357).

### TNF-Related Apoptosis-Inducing Ligand (TRAIL)

TNF-related apoptosis-inducing ligand, a member of the superfamily of TNF ligands, is a homotrimeric type II TM protein with a conserved C-terminal extracellular domain that mediates receptor binding and which can be cleaved by metalloproteinases to generate a soluble mediator (390). TRAIL is produced by several cell types, including immune cells such as macrophages and T cells and can be induced by both type I and type III Interferons (IFNs), a family of cytokines with fundamental importance in the innate immune response to viral infections (209, 391). Macrophages generate both soluble and membrane-bound TRAIL, which operate through distinct receptors on infected and non-infected, neighboring cells (209). TRAIL is a potent activator of cell death in transformed cells and activates cellular stress pathways in epithelial cells, as such finally leading to caspase-dependent or -independent cell death (209). In view of the prominent role of IFNs in antiviral response, IFN-dependent induction of TRAIL is a prominent regulator of disease outcome especially in respiratory viral infection, enters into the scene (209). As such, the IFN/TRAIL signaling axis is of potential interest in disease progression and attenuation of tissue injury during respiratory viral infection (209). Here we focused on the role of TRAIL in edema reabsorption and in alveolar epithelial function.

#### TRAIL Disrupts the Alveolar Epithelial Barrier

TRAIL plays adverse roles in viral infection (392–394). On the one hand, TRAIL drives infected cells into apoptosis in order to limit virus distribution (209). On the other hand TRAIL can induce functional and structural damage not only in infected cells, but also in bystander cells, such as uninfected cells of the alveolar epithelium (199, 208). As such TRAIL can at the same time prevent viral spreading, but also cause lung injury in acute respiratory viral infection (209). Accordingly, in influenza A virus (IAV) infection, TRAIL acts as a detrimental factor contributing to tissue injury and impaired inflammation resolution when released in excessive amounts by recruited immune cells (209). The activation of proapoptotic and pro-necroptotic pathways in respiratory infection can result in a structural disruption of the airway and the alveolar epithelial barrier, which is a major hallmark of respiratory disease and its progression to the ARDS (395, 396).

#### TRAIL Decreases Na+/K+-ATPase Expression and Impairs AFC

Peteranderl et al (199). have investigated whether IAV infection alters Na+/K+-ATPase expression and function in AECs and the ability of the lung to clear edema. IAV infection reduced α1 Na+/K+-ATPase expression in the plasma membrane of human and murine AECs and in distal lung epithelium of infected mice. Accordingly, the decreased Na+/K+-ATPase expression impaired AFC in IAV-infected mice. A paracrine cell communication network between infected and non-infected AECs and alveolar macrophages was identified, which led to decreased alveolar epithelial Na+/K+-ATPase function, thus to AFC inhibition (199). The IAV-induced reduction of Na+/K+-ATPase was mediated by a host signaling pathway that involved epithelial type I IFN and an IFN-dependent elevation of macrophage TRAIL (199). In non-infected cells within the IAV-infected lung, TRAIL severely compromised the function of the ion channel Na+/K+-ATPase, which was mediated by induction of the stress kinase AMPK (199) thereby potentially revealing a cross-link to TRAIL-induced autophagic cell stress pathways in bystander cells both *in vitro* and *in vivo* (199). The TRAIL-induced and AMPK-mediated downregulation of the Na+/K+-ATPase, a major driver of vectorial ion and fluid transport from the alveolar airspace toward the interstitium, resulted in a reduced capacity of IAV-infected mice to clear excessive fluid from the alveoli (395). Thus, TRAIL signaling contributes to intensive edema formation, a hallmark of disease in virus-induced ARDS (395). Notably, this effect of TRAIL on Na+/K+-ATPase expression was induced independently of cell death pathways elicited by caspases, as treatment of cells and mice with a specific caspase-3 inhibitor diminished apoptosis in AECs but still allowed for the reduction of the Na+/K+-ATPase (199).

### Transforming Growth Factor-β (TGF-β)

Transforming growth factor-β is a pleiotropic cytokine with a broad regulatory role in the immune system. Three highly homologous isoforms - TGFβ1, TGFβ2, and TGFβ3—share a receptor complex and signal transduction pathway, but their tissue expression levels are different (397). All are produced as inactive complexes, which must be activated to bind to their receptors (398). Platelets, T lymphocytes, macrophages, ECs, keratinocytes, smooth muscle cells, fibroblasts, i.e., a wide range of cells, can produce TGF (399). Following wounding or inflammation, all these cells are potential sources of TGF-β (400). Receptors for TGF-β have been found almost on every cell type tested so far, which enables this cytokine to exert its effects on almost any body tissue (401). Classically, TGF-β receptor signaling occurs by activating the Smad-dependent intracellular signaling pathway (398). The TGFβ receptor complex consists of two receptor subunits, TGF-β receptor (TGF-βR) I and II (398). These receptors mediate multiple responses (401).

#### TGFβ Context-Dependent Mode of Action

Transforming growth factor-β action is highly context-dependent and can be influenced by cell type, culture condition, interaction with other signaling pathways, developmental or disease stage *in vivo* and innate genetic variation among individuals (402). As such, TGF-β can be both a pro- and anti-inflammatory cytokine, which affects the growth and proliferation of many cell types (399). During inflammation, TGF-β1 is also able to effectively inhibit inflammatory response (403). The action of TGF-β following inflammatory responses is characterized by increased production of extracellular matrix components, as well as mesenchymal cell proliferation, migration, and accumulation (404). Pleiotropic nature of TGF-β modulates expression of adhesion molecules, provides a chemotactic gradient for leukocytes and other cells participating in an inflammatory response in one hand and, in contrast, inhibits them once they have become activated (405). Also in autoimmunity, TGFβ represents a double-edged sword (406). It can cause both T-cell growth promotion, as well as immune suppression (406).

#### Role of TGF-β Role in Pulmonary Edema

Transforming growth factor-β has a dual role in pulmonary edema. It can up- or downregulate alveolar ion and fluid transport, through its impact on ion channels/pumps (ENaC, CFTR and Na+/K+-ATPase) or on the pulmonary barrier. As such, TGF-β can decrease the expression of ENaC through decreasing expression of its α subunit mRNA and protein during bacterial infection (132). During ALI/ARDS, increased TGF-β1 activity in the distal airspaces promotes alveolar edema by reducing distal airway epithelial sodium and fluid clearance (132). Moreover, TGF-β can induce the internalization of βENaC from the lung epithelial cell surface and, hence, block the sodium-transporting capacity of AECs (133). In fact, TGF-β causes the subsequent activation of phospholipase D1, phosphatidylinositol- 4-phosphate 5-kinase 1α, and NADPH oxidase 4 (Nox4) (133). Nox4 activation moreover results in the production of ROS, which in turn reduces cell surface stability of the αβγENaC complex and thus leads to edema fluid accumulation (371). Apart from its effects on ENaC expression, TGF-β can also decrease CFTR expression and function (179) and it, moreover, impairs expression of the Na+/K+-ATPase β1 subunit, resulting in decreased Na+/K+-ATPase activity in lung epithelial cells (197, 198).

Transforming growth factor-β decreases lung epithelial barrier function (203–205) *in vitro* by a mechanism that involves depletion of intracellular glutathione (206, 407). The cytokine moreover induces endothelial barrier dysfunction *via* Smad2-dependent p38 activation (235).

The integrin αvβ6 (408) can activate latent TGF-β in the lungs and skin (409). Using this clue, Pittet et al. have shown that mice lacking integrin αvβ6 are completely protected from pulmonary edema in bleomycin-induced ALI. Furthermore, pharmacologic inhibition of TGF-β also protected wild-type mice from pulmonary edema induced by bleomycin or *Escherichia coli* endotoxin (206). In short, integrin-mediated local activation of TGF-β is critical for the development of pulmonary edema in ALI, and blocking TGF-β or its activation attenuates pulmonary edema. This neutralization can be done e.g., by the administration of a soluble type II TGF-β receptor, which sequesters free TGF-β during lung injury (206).

All of the deleterious actions of TGF-β discussed above will ultimately lead to decreased ion transport and may, therefore, promote and worsen pulmonary edema. However, TGF-β can also positively impact pulmonary edema. Intriguingly, TGF-β was proposed to increase the function of ENaC, *via* enhancing the expression of Na+/K+-ATPase α1- and β1-subunits (134).

### Interleukin-8

Interleukin-8 is a pro-inflammatory chemokine produced by a variety of tissue and blood cells (410), including bronchial epithelial cells (411), that correlates with neutrophil accumulation in distal airspaces of patients with ARDS. IL-8 is also a predictor of mortality in ALI (412–414). As such, significantly higher concentrations of IL-8 are found in the pulmonary edema fluid and plasma of patients with a septic versus a non-septic etiology of ARDS (415). Moreover, IL-8 promotes edema formation by blocking AFC (105).

#### The Role of IL-8 in Inhibiting β2AR Agonist

**Figure 8:**
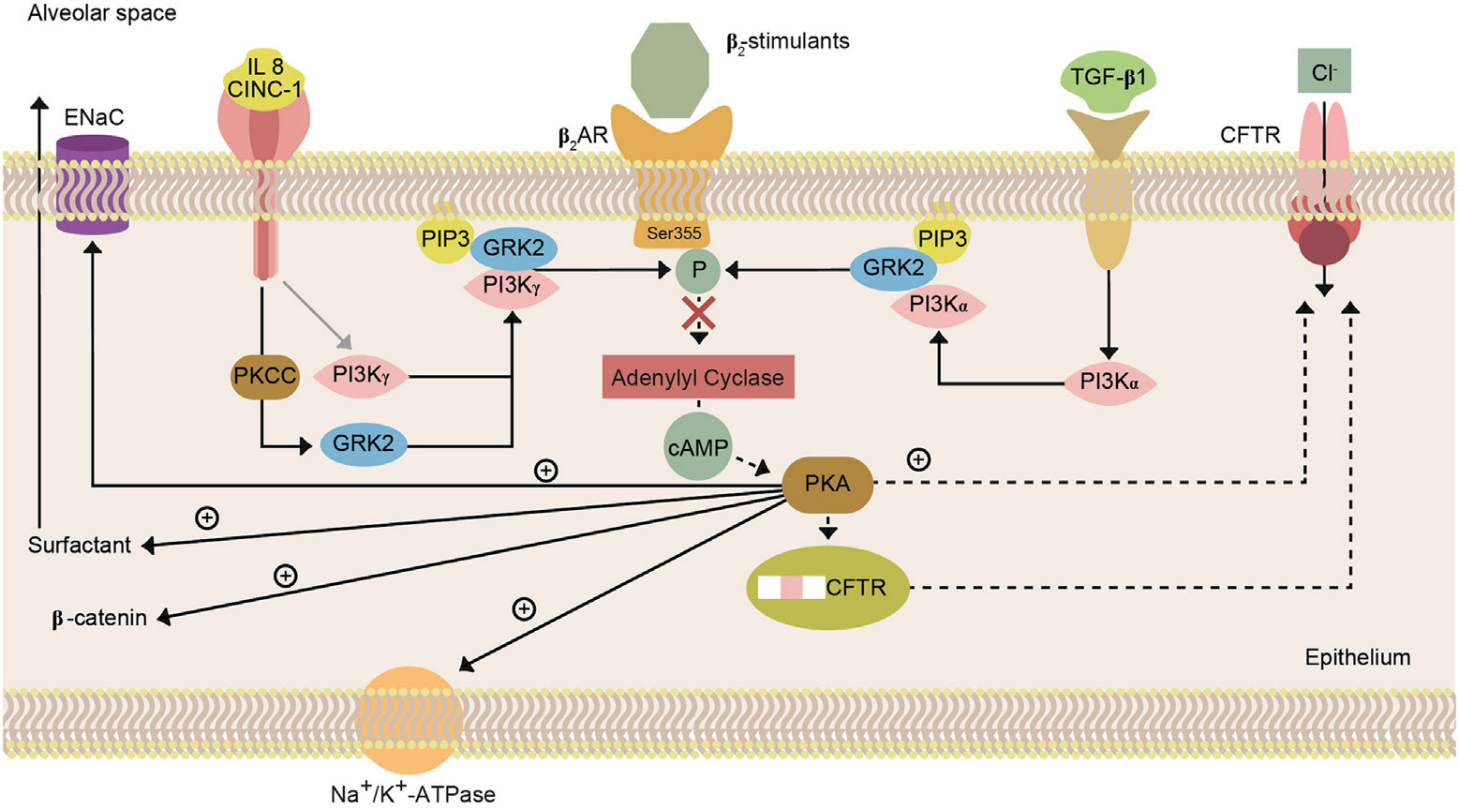
Rationale for the problematic role of β2 adrenergic agonists in clinical trials. Schematic representation of the mechanisms by which interleukin-8 (IL-8)/cytokine-induced neutrophil chemokine (CINC)-1 and transforming growth factor (TGF)-β1 have a synergistic inhibitory effect on the β2-adrenergic receptor (β2AR) signaling pathway in type II alveolar (ATII) cells. IL-8/CINC-1 and TGF-β1 cause the activation of different phosphoinositide 3-kinase (PI3K) isoforms. However, IL-8/CINC-1 but not TGF-β1 phosphorylates G-protein-coupled receptor kinase 2 (GRK2) *via* a protein kinase C-zeta (PKC-ζ)-dependent mechanism explaining why the blockade of IL-8/CINC-1 prevents the TGF- β1-mediated inhibition of the β2AR signaling pathway in ATII cells. This results in the translocation of the protein complex GRK2 and PI3K to the cell membrane. This protein complex causes phosphorylation at the Ser355 heterologous desensitization and downregulation of the β2AR in ATII cells. IL-8/CINC-1 and TGF-β1 then prevent the activation of 3′-5′-cyclic adenosine monophosphate (cAMP)/protein kinase A (PKA) pathway that upregulates the vectorial fluid transport across the alveolar epithelium *via* phosphorylation and increased expression of cystic fibrosis transmembrane conductance regulator (CFTR) at the plasma membrane of ATII cells. The solid lines indicate the pathways stimulated by IL-8/CINC-1 and the dashed lines indicate the pathways inhibited by these mediators (416).

Roux et al (105). have shown that IL-8 or its rat analog cytokine-induced neutrophil chemokine-1 significantly decreased β2AR agonist-stimulated vectorial Cl^−^ and net fluid transport across rat and human alveolar epithelial type II cells, through reducing CFTR activity and biosynthesis (105). This reduction process was mediated by heterologous β2AR desensitization and downregulation (50%) *via* the G-protein-coupled receptor kinase 2 (GRK2)/PI3K signaling pathway (105) (Figure 8). Consistent with the experimental results, high pulmonary edema fluid levels of IL-8 (>4,000 pg/ml) were associated with impaired AFC in patients with ALI. Taken together, these results suggest a role for IL-8 in inhibiting β2AR agonist-stimulated alveolar epithelial fluid transport *via* a GRK2/PI3K-dependent mechanism (105). On top of this, IL-8 can promote edema formation by increasing endothelial permeability (250).

### Interleukin-1β

Interleukin-1β is associated with decreased alveolar fluid reabsorption and thus with worse outcome in ALI and sepsis. IL-1β primarily decreases alveolar fluid reabsorption *via* a p38 MAPK, reducing the expression of the α-subunit of ENaC (113) as well as the β-subunit (137). In ARDS patients, the mean initial plasma levels of TNF IL-1β, IL-6, and IL-8 were significantly higher in non-survivors and in patients with sepsis. High plasma levels of IL-1β were associated with poor patient outcome (417). Likewise, high levels of IL-1β in the lungs of patients with ARDS were associated with an increased risk of mortality (417). The FAS/CD95 system acts together with TNF and IL-1β (57, 219, 418–420), leading to NF-ĸB production and neutrophil accumulating IL-8 secretion. Of note, an epithelial repair effect for type II pneumocytes *via* IL-1β was described in the injured alveolus (139), possibly in a specific context of cytokines, mediators and growth factors (139). Only in a specific fetal context IL-1β may increase alveolar fluid reabsorption by a hypothalamus-pituitary-adrenal gland axis (421) and an increase of both ENaC and Na+/K+-ATPase expression (140).

### Fas/FasL System (CD95/CD95 Ligand System)

Fas is a 45-kDa type I cell surface receptor that belongs to the TNF receptor family. It can cause cytokine and chemokine release, especially the neutrophil attractant IL-8, *via* MAP kinase activation in lung epithelial cells, as such promoting inflammation (219). Binding of FasL to membrane Fas activates apoptosis through activation of caspases, which seems the key to AEC apoptosis, thus epithelial barrier breakdown and its consequences in ALI (57, 418).

### Keratinocyte Growth Factor (KGF, FGF-7)

KGF is an epithelial cell-specific growth factor that has been shown to exert beneficial actions in many animal models of ALI and ARDS as well as in the *ex vivo* human lung (143, 422–430). Rats in which KGF was intratracheally administered increased AFC by about up to 50%, and this was further increased by the β2 agonist terbutaline (427). *In vitro* studies using mesenchymal stem cell-derived medium suggested that this growth factor plays a dominant role in tissue repair, even in the presence of the inflammatory cytokines IL-1β, TNF-α, and interferon-gamma, as well as in hypoxia. The observation that no downregulation of ENaC-α expression occurred despite of the presence of three key inflammatory cytokines suggested a dominant biological role of KGF in the acutely injured alveolar milieu (431). There is a currently a large interest in stem cell therapies as therapeutic approaches in clinical disorders like myocardial infarction, limb ischemia, diabetes, hepatic and renal failure, and ALI/ARDS. Stem and progenitor cell therapies as well as work with factors influencing those cells to reduce injury and increase repair have been performed. KGF has been proposed to be one of the main candidates to promote the repair capacity of stem cells in ALI. A recently performed double-blind, placebo-controlled phase 2 clinical trial—the KARE trial—tested the effects of KGF in 29 verum patients versus 31 placebo patients (432). There was no difference in the primary outcome variable, the oxygenation index, at day 7, and the treatment group had a trend to higher mortality, and more adverse events in terms of pyrexia. Nevertheless, these data do not exclude that the combined use of KGF and stem cells might provide protection in ALI.

### Soluble Receptor for Advanced Glycation End Products (sRAGE)

Receptor for advanced glycation end products, first characterized in 1992 by Neeper et al. is a 35 kDa TM receptor which belongs to the immunoglobulin superfamily (433). RAGE is one of the AT1 cell-associated proteins in the lungs (434, 435). RAGE and its ligands have been recognized to be involved in the pathobiology of a wide range of diseases which are accompanied by symptoms, like enhanced oxidative stress, immune/inflammatory responses, and altered cell functions (436). RAGE is highly expressed in the lungs at readily measurable levels and its level increases quickly at sites of inflammation, mainly in inflammatory and epithelial cells (437). RAGE has three forms, consisting of N-truncated, dominant-negative, and soluble RAGE, which can be produced either by natural alternative splicing or by the action of membrane-associated proteases (438). The correlation between sRAGE levels and AFC rate was investigated in both a clinical study of patients with ARDS, as well as in an experimental model of acid-induced lung injury in mice (264). The results obtained showed a correlation between elevated levels of sRAGE with lung injury and an impairment of AFC (264). Accordingly, an increase in alveolar–capillary barrier permeability, arterial oxygenation impairment, lung injury scores, and the extent of human lung damage on CT scan are all associated with sRAGE levels (264). Conversely, it has been shown that RAGE regulates lung fluid balance *via* protein kinase C-gp91(phox) signaling to ENaC (177). In fact, hAGE, a RAGE ligand, increases ENaC activity through oxidant-mediated signaling, which can ultimately impact lung fluid clearance (177).

## β2ARs as Important Modulators of AFC

### Structure and Subtypes

β2-adrenergic receptors are G protein-coupled receptors with seven-TM domains (439). Their three subtypes are β1, predominantly found in the heart, β2 in the respiratory system, and β3 in adipose tissue (440). β2 adrenergic agonists activate the β2-adrenoceptors (β2AR) on airway smooth muscle and are used to treat bronchoconstriction in asthma and chronic obstructive pulmonary disease (COPD) (441). In their canonical signaling pathway, agonist binding couples the β2AR to the Gs subtype of G protein. Gs activation leads to adenylyl cyclase, production of cAMP and activation of the cAMP-dependent protein kinase A (PKA), which mediates most of the functional consequences of Gs-coupled receptor activation (442). In airway smooth muscle, β2AR-stimulated PKA activity mediates relaxation through phosphorylation of multiple proteins involved in regulating intracellular calcium levels, calcium sensitivity, and cross-bridge cycling (442).

### The Role of β2AR Agonists in AFC

The presence of pulmonary β2ARs includes the alveolar space and provides the possibility to modulate the active Na^+^ transport. β2adrenoceptors and the β-adrenergic agonists accelerate AFC (439) due to Na^+^ transport *via* an amiloride-sensitive pathway (443) as shown *in vitro* (444), *ex vivo* (445), and *in vivo* in rat (446), dog (447), sheep (448), guinea pig (449), mouse (443, 450), and human lung tissue (451). β2AR knockout mice results suggest that the β2AR is responsible for most of the β-adrenergic–mediated upregulation of AFC (452). Therefore, β2ARs appear to be responsible for the bulk of the β-receptor–sensitive alveolar active Na^+^ transport likely due to direct and indirect up-regulation of the alveolar active Na^+^ transport (445, 449, 452–454). β-agonists *via* activation of β2ARs regulate necessary key proteins for the process of alveolar epithelial active Na^+^ transport such as ENaC, Na+/K+-ATPase and CFTR in animal models as well as in human lung tissue (445, 449, 453, 455). β2ARs mediate short-term regulation of Na^+^ pumps which occurs within minutes of receptor engagement *via* highly regulated recruitment of assembled Na+/K+-ATPase from intracellular compartments through phosphorylation of intermediary proteins and RhoA-kinase (456, 457). Long-term regulation is carried out *via* transcription (458) and translation of α1-subunit of Na+/K+-ATPase and ENaC subunits through PKA induced phosphorylation of cAMP-responsive elements and post-transcriptional regulation *via* mitogen-activated protein kinase/extracellular signal–regulated kinase and rapamycin sensitive pathways (455, 459) by direct modulation of Na^+^ channels at the apical surface of the cells (460) or an activation of PKA to modulate a cation channel (92, 453).

#### Impact of β2AR Agonists on ENaC

Protein kinase A-mediated β2-agonist action phosphorylates cytoskeleton proteins and promotes trafficking of Na^+^ channels through the cell membrane and direct phosphorylation of epithelial Na^+^ channel β and γ subunits stimulate the β2AR and increases the number of epithelial Na^+^ channels and their open time in alveolar type II cells (453) and enhances the expression of the α-subunit of the epithelial Na^+^ channel ENaC (458). β-agonists and cAMP analogs increase the open probability and open time of amiloride-sensitive Na^+^ channels (161). β2AR agonists thus increase Na^+^ flux across the apical cell membrane by increasing both membrane-bound channel abundance and Na^+^ flux through ENaC (439).

#### Impact of β2AR Agonists on Na+/K+-ATPase

β-adrenergic agonist modulate Na+/K+-ATPase partially through adenosine 3’,5’-cyclic monophosphate (461). β2-adrenergic agonists increase the gene expression of Na+/K+-ATPase which leads to:

- Increased expression of α1-Na+/K+-ATPase mRNA and protein (458).
- Increase of the quantity of Na+/K+-ATPase (458)
- Increased activity of Na+/K+-ATPase (456, 458, 462–464).

#### Impact of β2AR Agonists on CFTR

Cystic fibrosis transmembrane conductance regulator is required for cAMP-mediated upregulation of fluid clearance, but is not necessary for basal fluid absorption (183), thus for alveolar fluid homeostasis in the uninjured lung (182, 183). β2-adrenergic stimulation activates CFTR by cAMP and PKA activation (184). In airway epithelial cells, the interaction of β2-AR with CFTR is mediated by scaffold proteins, such as NHERF1, allowing its interaction with PKA and stabilizing it on the plasma membrane (465). β2-adrenergic stimulation increases CFTR regulator expression in human airway epithelial cells through a cAMP/PKA-independent pathway (466).

### β2-Adrenergic Agonists Are at Least in Part Not of Clinical Benefit in ALI/ARDS Studies and May Increase Mortality

In mild-to-moderate lung injury, alveolar edema fluid clearance is often preserved by catecholamine-dependent or -independent mechanisms (467). Stimulation of AFC is then related to activation or increased expression of sodium channels like ENaC or the Na+/K+-ATPase pump and may involve CFTR (467). In severe lung injury, AFC perturbation result through increased endothelial-interstitial-epithelial alveolar permeability and changes in activity or expression of sodium or chloride transport molecules (467). Improved barrier function and increased alveolar fluid reabsorption, theoretically by β-adrenergic agonists or the lectin-like TNF activity or alternatives, vasoactive drugs, regenerative or repair measures are therefore therapeutic alternatives (467). Whereas in the BALTI-2 study with salbutamol given as an intravenous infusion for up to 7 days, compared with a placebo, more than 160 patients [age 55 (SD 17) years] per group were studied, the study was stopped as salbutamol treatment was associated with increased 28-day mortality of 34% compared to 23% (risk ratio 1.47, 95% confidence interval 1.03 to 2.08) (468).

Salbutamol early in the course of ARDS was poorly tolerated. The authors concluded that such a β2-agonist therapy is unlikely to be beneficial and could worsen outcomes. Follow-up data further suggested worse outcome at 6 and 12 months in ARDS patients treated with salbutamol. They discussed that further trials of β-agonists in patients with ARDS were therefore unlikely to be conducted.

Some questions remained open, such as whether or not there may be benefit at a different dose or in specific populations (468). The survival curves for salbutamol and placebo appeared to continue to diverge after the end of the study drug infusion after 7 days, suggesting that the mechanisms may involve indirect effects as, e.g., more systemic disease under and after intravenous salbutamol. Concerning morbidity and mortality, Salbutamol can cause arrhythmia and tachycardia, and electrolyte and metabolic disturbances such as hypokalemia, hypomagnesemia, and lactic acidosis, which was observed in the study, and led to more salbutamol discontinuation. The used salbutamol dose of 15 µg/kg ideal body weight/hour i.v. was considered the maximum that critically ill patients could receive without an increase in ventricular or atrial tachycardia or ectopy. It was at the higher end of the recommended dosing regimen, and it is possible that lower doses might have been better tolerated and caused fewer adverse outcomes (468).

Rather similar results were observed in the USA in the ALTA trial (Albuterol for the Treatment of ALI). ALTA was a placebo-controlled multicentre study of nebulized salbutamol in patients with ALI. Patients were randomized to receive either salbutamol 5 mg every 4 h or saline placebo, for up to 10 days. The primary outcome was ventilator-free days. Recruitment started 2007 with a target sample size of 1,000 patients. It was terminated after 282 patients had been enrolled because of futility. There was no clear difference observed in both ventilator-free days between the salbutamol and placebo arms (14.4 versus 16.6 days; 95% CI –4.7 to 0.3 days) or in hospital mortality (salbutamol 23.0% versus placebo 17.7%; 95% CI –4.0% to 14.7%). Although the β2 stimulator intervention was delivered by a different route in ALTA, and the early termination of recruitment caused that confidence intervals are wide, the results seemed much consistent with the BALTI-2 trial.

One alternative way was to use combination of inhaled corticosteroid and inhaled β2 agonist. In a recently published pilot study, a typical asthma treatment combination of twice daily inhaled formoterol and budesonide for 5 days showed its feasibility and promising results. The rationale was to reduce by both budesonide and formoterol alveolar inflammation, and to further improve by formoterol AFC. The aim was to reduce ARDS. More patients in the placebo group developed ARDS (7 versus 0) and required mechanical ventilation (53% versus 21%) (469).

### Further Potentially Critical Mechanisms of Action β-Adrenergic Agonists

Besides two futile ARDS trials, further factors might restrict the β2 receptor agonist usage as a therapy to increase the resolution of pulmonary edema (467). Prolonged stimulation of β-adrenergic receptors with endogenous catecholamines could desensitize the β-receptors and prevent their stimulation with exogenous catecholamines (467). For instance, in some patients the alveolar epithelium might be too injured to respond to β-adrenergic agonist therapy (467), likewise circulating factors could limit the action of β-adrenergic agonists (467). Also, in the presence of left atrial hypertension, atrial natriuretic peptide can inhibit the stimulatory effect (467). Similarly in prolonged hemorrhagic shock and resuscitation, cAMP agonists may not stimulate AFC because oxidant-mediated injury may reduce the response of the alveolar epithelium to β-2 agonists (467).

An important clinical aspect is the potential to increase cardiac index by β2 receptor agonists (470), by both cardiac stimulation and pulmonary arterial vasodilation. Cardiac stimulation can lead to a higher cardiac index. This is potentially dangerous, as due to the injured lung put in the circulation in series, there is an increase in filtration, which further increases alveolar fluid and gas exchange disturbance. An interrelated second, and in ALI most probably untoward “Robin Hood effect” of potential opening of vascular beds that are closed by vasoconstriction is, e.g., observed in COPD patients inhaling β2 receptor agonists and developing more hypoxemia (471). This is probably due to increased perfusion in badly ventilated ALI/ARDS alveolar areas. As shown by Briot et al., β2 receptor agonist therapy seems therefore to have the potential to heighten the protein leakage from plasma to alveoli in the acutely injured lung (470).

## Protein Clearance Out of the Alveolar Space

Clearance of serum and inflammatory proteins from the alveolar space is an important and possibly vital process in recovery from pulmonary edema. Albumin and IgG are present in pulmonary edema fluid in concentrations that are 40–65% of plasma levels in hydrostatic pulmonary edema and 75–95% in non-cardiogenic pulmonary edema. Concentrations of albumin, for example, may be 5 g/100 ml or more. Protein concentrations rise during recovery from alveolar edema because the salt and water fraction of edema fluid is cleared much faster than albumin and IgG. Clearance of alveolar protein occurs by paracellular pathways in the setting of pulmonary edema. Transcytosis may be important in regulating the alveolar milieu under nonpathological circumstances. Alveolar protein degradation may become important in long-term protein clearance, clearance of insoluble proteins, or under pathological conditions such as immune reactions or ALI.

Early since the first descriptions of ARDS, we know that protein content is high, “haemorrhagic,” and about the same as plasma proteins. Plasma and coagulative products such as fibrin strands are degraded or modified, e.g., also to hyaline membranes in a high number of patients (31). They are observed in ARDS, are especially covering denuded basement membranes where pneumocytes are missing, and may be related to adverse outcome (56).

Recent research hints to a better understanding of the resolution of those alveolar proteinaceous contents and debris out of the distal airways. Counterintuitively, neither macrophages, nor the mucociliary transport processes seem to play major roles in protein clearance also over several days time (472). Protein clearance from the distal air spaces is in part facilitated by active endocytotic processes including for albumin by the 600 kDa TM glycoprotein called megalin or LDL-receptor related protein-2, a member of the low-density lipoprotein-receptor superfamily (6). Again, its important functional inhibition seems TGF-beta1 related. Megalin seems negatively regulated by glycogen synthase kinase 3b (GSK3b). An important regulator for this protein kinase signaling molecule seems the RNA binding protein Embryonic Lethal, Abnormal Vision, Drosophila Like 1/Human antigen R (ELAVL-1/HuR) as an upstream regulator of GSK3b (6). ELAVL-1/HuR is an RNA binding protein that increases mRNA stability. Its importance has been shown in ventilator-induced and acid-induced mouse lung injury. In EC lines it induces ICAM-1 and IL-8 after TNF stimulation.

Endocytosis of macromolecules can be mediated by a non-selective fluid phase uptake, which is a very slow process in alveolar epithelium. A receptor-mediated endocytosis is much faster and occurs when specific high-affinity receptors are implicated. Two pathways are described, called caveolae-mediated and clathrin-mediated endocytosis.

Detailed research on alveolar protein and debris clearance have only recently begun. Judging their roles is more complex, as hyperosmotic stimuli might be of anti-inflammatory action, and possibly there is even more biological signaling as formerly assumed that may influence underlying lung disease.

## Potential Novel Approaches to Understanding the Effects of Ion Channel Stimulants in Lung Disease

### Hyperosmolarity, High Na^+^ Content, or High Oncotic Pressure

One biological effect that has, to our knowledge, not yet been assessed is the question whether due to fluid reabsorption out of the alveolus the hyperosmolarity or hyper-oncotic situation is of biological effect. Several limitations have to be mentioned: Certainly the pulmonary surfaces including the mucus and the surfactant system and its layers are complex and disease-prone systems, as suggested in cystic fibrosis. Dose- and time response have to be taken into account. Actually, there are contradictory results on those effects: Some observations described anti-inflammatory effects of hyperosmolarity in the airways, as in the nose and sinuses with a few randomized controlled trials that compared isoosmotic versus hyperosmotic irrigating solutions (473, 474). Honey is hyperosmotic and antibacterial, and in wound healing it seems frequently beneficial (475). This is also the case for hyperosmotic salt pastilles in throat and neck infections. However, nebulized hypertonic saline is still disputed in infants with acute viral bronchiolitis (476). There are also *in vitro* cell model results showing a switch from adaptive to inflammatory gene expression by hyperosmotic stress by protein kinase R activation, NF-kappaB p65 activation with responsive genes including inducible NO synthase, interleukin-6, and interleukin-1β (477), others with some protection *via* p53 gene regulation (478). In a rat seawater drowning model, alveolar hypertonicity, but not iso-or hypotonicity-induced inflammation and vascular leak, thus edema probably by hypoxia-inducible factor-1 and including ataxia telangiectasia mutated kinase and PI3 kinase (479).

Local Na^+^ accumulation and enhanced availability have been linked to activation of tonicity-responsive enhancer binding protein (TonEBP) *via* the mononuclear phagocyte system in the skin (480), a system also widely represented in the lung. Enhanced local Na^+^ has been shown to boost pro-inflammatory TH17 cell production and, finally, IL-17 release (481). The pro-inflammatory phenotype is maintained in high-salt conditions with upregulation of TNF-α and IL-2. As it is currently unclear what is the mechanism of enhanced Na^+^ presentation to activate the TonEBP, an enhanced Na^+^ accumulation in the extracellular matrix (482), the activation of Na^+^ channels or even a permissive role of an altered Na+/K+-ATPase activity *via* endogenous ouabain have to be considered (483). As all of these mechanisms are also represented in the lung, both Na^+^ presentation and availability should, therefore, be considered in pulmonary fluid regulation.

Briefly, there may be important, but so far not yet well understood anti-, or even pro-inflammatory, stimuli, or signals by hyperosmotic stimulation, underlining the importance to investigate this subject further.

## Specific Clinical Settings with Potential Significance of Alveolar Fluid Reabsorption in Inflamed Lungs

### RDS in the Newborn

Respiratory distress syndrome is one of the most important causes of morbidity and mortality in newborns and has a prevalence of about 1%. It is clinically manifesting as respiratory distress accompanied by abnormal pulmonary function and hypoxemia directly in the first minutes or hours after birth. RDS prevalence increases with decreasing gestational age. As such the incidence of RDS is highest in extremely preterm infants, affecting more than 90% of infants at a gestational age of 28 weeks or less. In a birth cohort of more than 230,000 deliveries, the syndrome was observed at 34 weeks gestation in 10.5%, at 35 weeks in 6%, at 36 weeks in 2.8%, at 37 weeks in 1%, at 38, and more in 0.3%. Therapy is supportive, includes surfactant replacement, fluid restriction, and glucocorticoids. Whereas a viewpoint has been that qualitative and quantitative surfactant deficiency, inflammation including alveolar neutrophil influx, and fluid overload (in part by low urine output) account for this syndrome, some reports hint to a suboptimal Na^+^ transport. During gestation, the lung epithelium secretes Cl^−^ and fluid and develops the ability to actively reabsorb Na^+^ only during late gestation. At birth, the mature lung switches from active Cl^−^ and consecutive fluid secretion to active Na^+^ and consecutive fluid absorption in response to circulating catecholamines. Changes in oxygen tension augment the Na^+^-uptake capacity of the epithelium and increase ENaC gene expression. The inability of the immature fetal lung to switch from fluid secretion to fluid absorption results, at least in large part, from an immaturity in terms of low expression of ENaC, where all three ENaC subunits are low in preterm relative to full-term infants. ENaC-α is increased in the respiratory epithelium by therapeutic glucocorticosteroids (484, 485).

However, in the last years the incidence of near-term and term infants with RDS has increased, and their clinical characteristics differ from those of premature infants with RDS. Li et al. found that death was virtually inevitable for some babies, despite intensive care and surfactant replacement therapy, particularly in near-term and term infants. Lung tissue slices taken during autopsies of near-term and term infants who died of neonatal RDS showed that some alveoli were obviously dilated, with a large amount of lung fluid. This was in addition to an alveolar collapse from a lack of surfactant, and suggested that lung fluid absorption disorders might be an important additional cause of RDS by influencing gas exchange or surfactant function (486). In their study on 120 neonates with RDS and 129 controls, 7 newborns died despite of intensive care and surfactant replacement therapy. All of them received surfactant more than once and four of them were near-term or term infants. Preterm babies (less than 35 weeks of gestational age) had a better response to surfactant treatment than near-term and term babies. These results were consistent with the finding that the surfactant therapy was not effective for all newborns with RDS. The authors assessed the relationship between RDS and 7 candidate polymorphisms of the *SCNN1A* gene that encodes α-ENaC. One single nucleotide polymorphism (rs4149570) of the *SCNN1A* gene was associated with RDS. Moreover, in a group of term infants (gestational age was 37 weeks or greater), another single nucleotide polymorphism locus (rs7956915) was associated with RDS. These results are consistent with the hypothesis that the causes of RDS are multifactorial, and that in term infants it might differ from those in preterm infants (487). Alveolar fluid reabsorption and, thus, α-ENaC might play a key role in the pathogenesis by influencing the amount of lung liquid absorption, especially in term infants with RDS.

### Acute Infection-Related Respiratory Failure

Pulmonary infections are the most prevalent infections worldwide, most of bacterial or viral origin. Community-acquired pneumonia is a frequent infectious respiratory disease with an annual incidence of about 5–12/1,000, and leads to hospitalization in 20–50% of patients. Mortality in hospitalized patients ranges from 5 to 15%. The most common reason for hospital admission in childhood is pneumonia and accounts for up to 50% of admissions. The high morbidity, mortality, and epidemiologic dangers with viral or bacterial pneumonias are of high concern. Pneumonia mortality is typically caused by flooding of the pulmonary alveoli preventing normal gas exchange and consequent hypoxemia. We refer to excellent recent reviews (117, 371). Of note is that pneumonia and sepsis are by far the leading causes of ALI and ARDS. Sepsis is a major healthcare burden, mirrored by up to 45% of intensive care unit costs (64) and bearing a high mortality of about 30%. Cytokines and ion channels are key elements in this common health problem.

### Lung Transplantation

Lung transplantation is a substitutive treatment of various end-stage pulmonary disease. Cystic fibrosis, COPD, and idiopathic pulmonary fibrosis (IPF) are the most important transplanted patient groups (488). The high mortality rate relative to other solid-organ transplants is in part due to chronic rejection. The limited availability of donor lungs results in a highly limited treatment strategy for patients in whom a survival benefit—estimated 5-year survival is about 60%—is expected (488).

**Figure 9:**
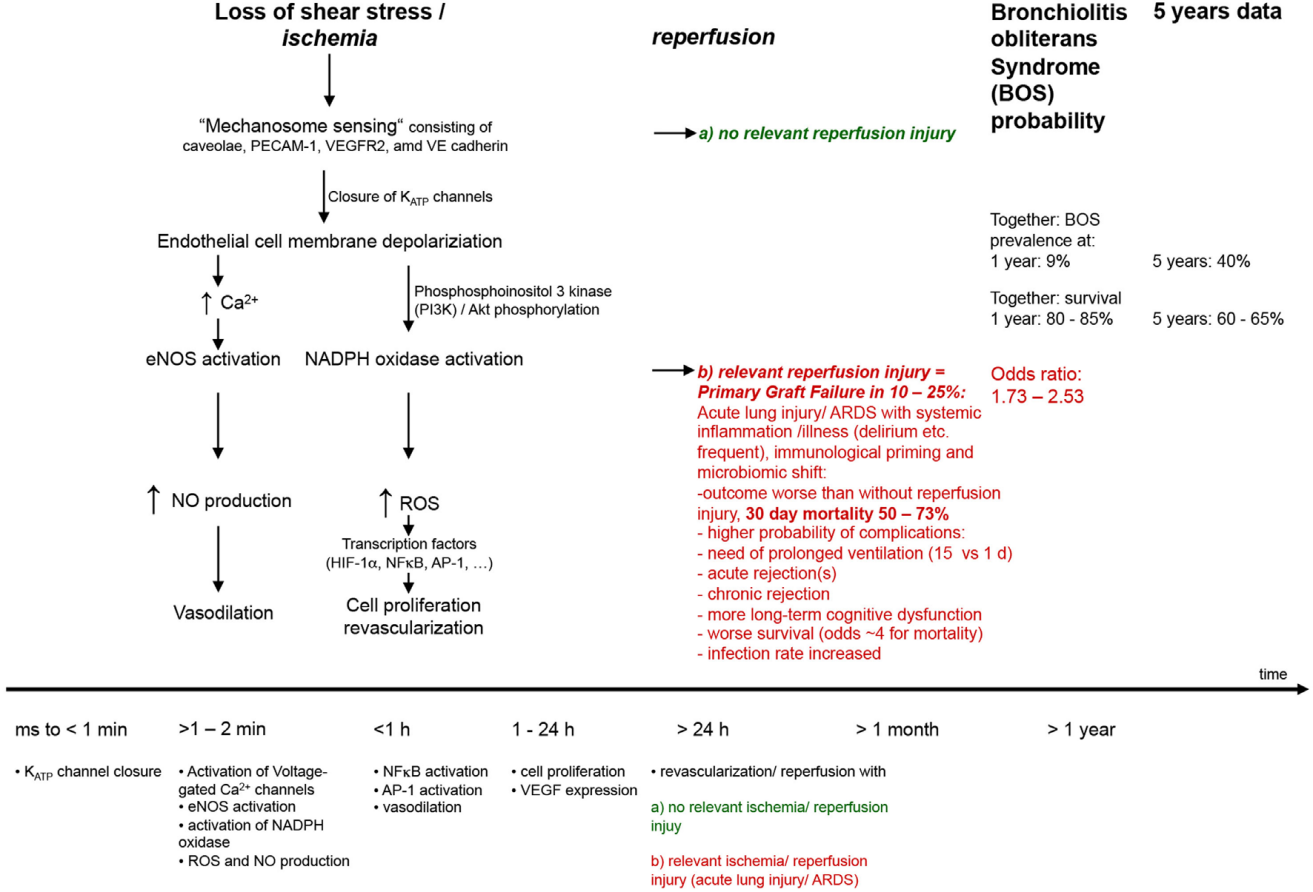
Pathophysiology and biological significance of pulmonary reimplantation response after lung transplantation (491–501). Adapted from Chatterjee et al (499), Whitson et al (496), Hartert et al (501), Basseri et al (498), Bharat et al (495), Bharat et al (497), Christie et al (492, 493), Huang et al (502), and King et al (491).

**Figure 10:**
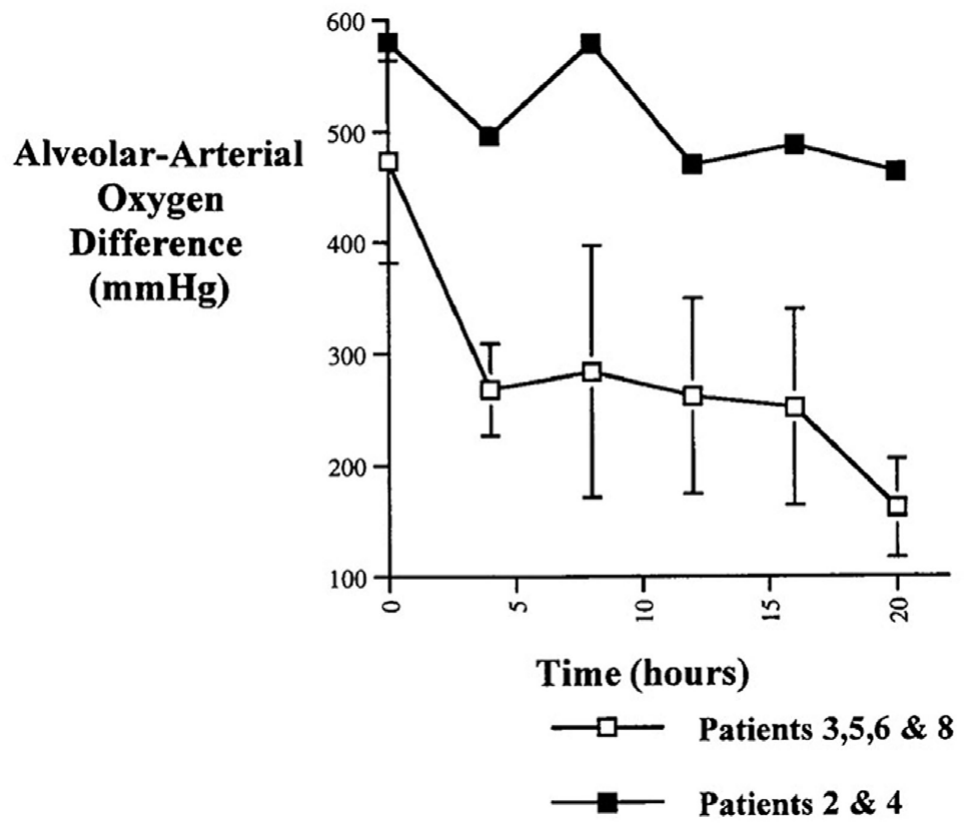
Time course of mean AaPO2 after the onset of reperfusion pulmonary edema. Comparison of mean AaPO2 in four patients with intact alveolar epithelial fluid clearance (open squares) to the patients with no net alveolar epithelial fluid clearance (solid squares). The data for Patients 3, 5, 6, and 8 are expressed as mean 6 SD. The data for Patients 2 and 4 are expressed as the average of the AaPO2 at each time point (45). Reprinted with permission of the American Thoracic Society. Copyright © 2017 American Thoracic Society.

Primary graft dysfunction (PGD) is termed the development of allograft infiltrates within 72 h of transplantation together with impaired oxygenation, when other identifiable insults such as volume overload, pneumonia, acute rejection, atelectasis or vascular compromises are excluded. PGD is usually referred to ischemia–reperfusion injury, but additionally to any further mechanical, surgical or chemical trauma such as inflammatory, neural or hormonal events of the donor, high oxygen fraction during reperfusion, or lymphatic disruption. PGD is mild and transient in most cases, but 10–20% of patient situations are sufficiently severe to cause life-threatening hypoxemia similar to ARDS, based on the same mediators and cytokines and a diffuse alveolar damage resembling ARDS. Similar to ARDS, it is considered a systemic disease, not only affecting the lung, but the whole patient. Thereby, the increased occurrence of cerebral dysfunction, i.e., patient delirium, worsens prognosis. Severe PGD quadruples perioperative mortality, the leading cause of early death of lung transplant recipients. In one study it is associated with a 30 day mortality of 63 versus 9%, and associated duration of mechanical ventilation is 15 versus 1 day (489) (Figures 9 and 10). The risk of higher morbidity and death risk persists even after an often protracted recovery, suggesting that PGD triggers an increased risk for bronchiolitis obliterans syndrome as a manifestation of chronic allograft rejection (490).

Ischemia–reperfusion injury is the main mechanism for PGD (503, 504). With logarithmic function ischemia time is associated with reperfusion injury: Whereas 4 h ischemia is associated with about 13% more risk than 2 h, 6 h ischemia increases the risk by more than 50%, 8 h by a factor of 3, and 10 h by a factor of about 8 (494). The hypothermic preservation increases oxidative stress, leads to accumulation of intracellular sodium and loss of intracellular potassium and an intracellular calcium overload, cell death with apoptosis (240) and necrosis. The release of pro-and anti-inflammatory cytokines such as TNF, INF-γ, IL-8, IL-10, IL-12, and IL-18 and complement cause smooth muscle contraction and increase vascular permeability, amplify by C5a the inflammatory response and are chemoattractant. Soluble complement receptor-1 is an accepted, but underused treatment based on a placebo-controlled clinical trial with 59 patients (505).

A huge part of the ischemia–reperfusion injury of lung allografts is mediated by the change in vascular shear stress due to the blood flow cessation. The endothelial sensing mechanism called mechanosome chiefly consists of PECAM-1, VEGR receptor-2 (VEGFR2) and VE cadherin in the EC caveolae (499). It closes the K_ATP_ channel of the EC membrane, depolarizes it and leads to NADPH oxidase 2 activation as the main source to generate ROS. EC depolarization results in opening of T-type voltage-gated Ca^2+^ channels, increase intracellular calcium, and NO synthase activation and consecutive NO-mediated vasodilation, and an overproduction of ROS that causes oxidative injury which triggers inflammation or even cell death (499). PI3K-Akt leads to NADPH activation, producing ROS. With ischemia, there is also an NO production by endothelial NO synthase, probably as a physiological response to the loss of blood flow. The ROS generated in ECs interact with signaling-related proteins and thus with enzymatic activity. NF-ƙB, activator protein 1 (AP-1), and c-Jun and c-Fos and the redox-sensitive HIF-1α, Nrf2, ATR/CREB are increased (499). Even although PMN are then recruited into lungs, the production of ROS by the endothelium is the initial signal.

Reperfusion further activates NADPH oxidase-2 leading to lipid peroxidation, which can be several fold more extensive than ischemia alone. Opening of an inward K^+^ channel was accompanied with hyperpolarization and ROS as well as NO production. Mainly a PMN influx and macrophage activation contribute to that injury. There is a strong correlation between excessive oxidative stress markers and the acute donor lung injury extent, and immunological rejection including later chronic rejection in terms of bronchitis obliterans syndrome as both the major causes for lung graft failure (499) (Figure 9).

The success of lung transplantation is much tempered by the limited organ supply. Many potential recipients are dying on the waiting list or being removed from the list because of clinical decline (506). Groups have therefore tried to expand the donor polls using extended criteria donors, with efforts to suggest rates of PGD, bronchiolitis obliterans syndrome, early morbidity and mortality to have equivalent to those with standard criteria donors (506). Most lung grafts come from brain-dead donors, but only about 15–20% of donors provide lungs that are satisfactory for lung transplantation (506). Strategies to expand the donor pool include the use of donation after cardiocirculatory death by doing a normothermic *ex vivo* lung perfusion (507), resulting in a study an about 28% increase in lungs suitable for transplantation. Problems are the increased risk of perioperative hypotension, warm ischemia time, a higher rate of aspiration, and more uncertainty to predict the lung’s usability for transplantation. *Ex vivo* assessment and reconditioning might overcome some issues in the longer term (506) (Figure 9).

**Figure 11:**
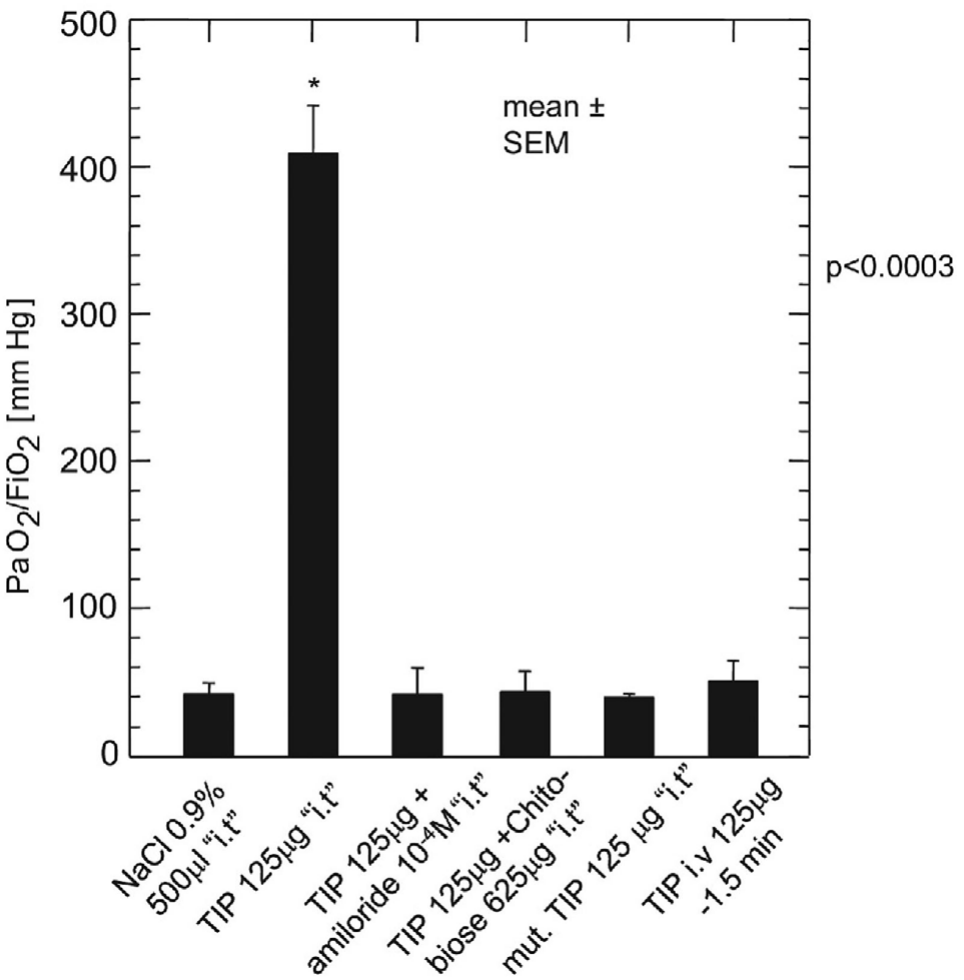
Oxygenation at 24 h after transplantation. At sacrifice, 24 h after reperfusion of the left-sided lung transplant, the PaO2/FIO2 ratio was measured after excluding the native right-sided lung by clipping the right-sided stem bronchus and right-sided pulmonary artery. The animals were tracheotomized and ventilated with an FIO2 of 1.0. The tumor necrosis factor tip peptide significantly increased gas exchange compared with all other study groups. *p < 0.003 versus NaCl. Data are mean ± SEM. i.t., intratracheally (8).

As shown before, using TNF tip peptide as preventative strategy in the left-sided unilateral orthotopic rat lung transplant model of prolonged cold ischemia we could show important biological effects, as highly severe lung injury with blood gas parameters qualifying for severe ARDS could be virtually prevented by the activation of the TNF lectin-like region (8) (Figure 11). The clinical pilot study of Aigner et al. suggests relevant improvement during established PGD by the TNF tip peptide (357). Both studies underline the biological potential of the TNF lectin-like region, i.e., the cytokine’s ion channel activation, thus its potent modulation of ALI, and thus its potential effect to prevent untoward long-term effects.

### Interstitial Lung Disease, Especially Acute Exacerbation of Idiopathic Pulmonary Fibrosis (aeIPF)

Idiopathic pulmonary fibrosis is a chronic and progressive lung disease of unknown etiology that occurs primarily in adults in their 50s and 60s and higher. Annual incidence is about 7–16 cases per 100,000 in the USA and 0.2 – 7 per 100,000 in Europe. Prognosis is severe with a median survival of about 2–3 years after diagnosis (508).

Acute exacerbation of idiopathic pulmonary fibrosis is a highly important disease progression of high morbidity and an extremely high mortality of 50–90% (509). It is typically reported to have an annual incidence of 5–15 or more %, with a higher incidence in advanced disease, and is defined as an acute worsening of dyspnea and lung function without an identifiable cause. Intriguingly, aeIPF has quite similar clinical features and similar prognosis compared with non-idiopathic causes of acute respiratory worsening in IPF such as infection or aspiration. It is, therefore, debated whether etiologies are to be separated (509).

There is some similarity between aeIPF and ARDS. However, the biological backgrounds are even much less understood. Gene expression profiles mainly show primarily infections or overwhelming inflammatory etiology, but more epithelial injury and proliferation as main profile, including gene expression of CCNA2, alpha-defensin, and apoptosis. Histopathologically, diffuse alveolar damage seems frequently observed in aeIPF. This finding is similar to ARDS and also has systemic multiorgan disease consequences, as evidenced by autopsy findings (510).

A number of current pharmacotherapies are under investigation for the therapeutic challenge of aeIPF as reviewed by Juarez et al., but no substance, combination of substances, or treatment modality (such as non-invasive ventilation which seems beneficial) has demonstrated such a clear benefit to become a new standard of therapy. This leaves clinicians with polypragmatic, mainly supportive care. Novel approaches are actually developed concerning immune suppression including calcineurin inhibitors, rituximab, removal of immune cells and mediators by either therapeutic plasma exchange or haemoperfusions with polymycin-B immobilized fibers aimed to remove not primarily endotoxin, but also contributing cytokines, and maybe hemostasis modulating agents such as intravenous recombinant thrombomodulin (508). The option of modulating the inflammation and to protect barrier function with, e.g., the biological action of TNF tip region is actually conceptualized in this group of severely sick patients.

### Pre-eclampsia

Pre-eclampsia refers to the new onset of the combination of hypertension and proteinuria or of hypertension and end-organ dysfunction without or with proteinuria in previously normotensive pregnant women after at least 20 weeks of gestation. About 4–5% of pregnancies worldwide are complicated with pre-eclampsia, and first pregnancies are more frequently associated with this disease. Together with hemorrhage, thromboembolism, and cardiovascular disease, pre-eclampsia is one of the four leading causes of maternal death, accounting for 15% of them in the Western world. Prevalence is about 1 maternal death per 100,000 live births. When pre-eclampsia occurs, the fatality rate is about 6 per 10,000. Severe acute diastolic dysfunction in severe pre-eclampsia can lead to pulmonary edema in this patient group. Maternal and fetal/placental factors seem responsible, such as abnormal trophoblast invasion of the spiral arteria of the decidua and myometrium early in pregnancy, a suboptimal uteroplacental blood flow possibly leading to high oxidative placental stress, altering placental angiogenesis, poor feto-placental vasculature and abnormal vascular reactivity. Endothelial dysfunction can be caused by systemic anti-angiogenic signals by anti-angiogenic factors. Elevated levels of soluble fms-like tyrosine kinase 1 (sFlt-1; an inhibitor of vascular endothelial growth factor), reduced levels of placental growth factor (PlGF), and an increased sFlt-1: PlGF ratio have been reported both in women with established pre-eclampsia and in women before the development of pre-eclampsia (511). This is moreover accompanied by increased pro-inflammatory cytokine production, which in turn promotes renal and pulmonary barrier dysfunction and impaired ion channel activity. As a consequence, pulmonary edema is a severe feature of the disease. In this case, the edema can be multifactorial, due to left heart failure, and thus excessive pulmonary vascular hydrostatic pressure, to decreased plasma oncotic pressure, to capillary leak, or to iatrogenic volume overload (511, 512).

### High-Altitude Pulmonary Edema

About 100 million people live at altitudes greater than 2,500 m, about 15 million above 3,000 m, and some above 5,000 m (384). Most have developed the ability to live and reproduce at elevation as high as 5,000 m, but in some cases, develop chronic medical problems due to their high-altitude residence. At 5,500 m the pressure is about only half the normal. Furthermore, many lowlanders venture to high altitude for work and recreation. These more acute exposures also pose the hazards of acute altitude illness, e.g., in Colorado skiers in 15–40% of them with an incidence of HAPE then of 0.1–1%. The prevalence of HAPE depends on an individual’s susceptibility, the rate of ascent, the final altitude, but also heavy and prolonged exercise, and is higher in male. At altitudes of 4,500 m the prevalence is between 0.2 and 6%, and at 5,500 m between 2 and 15% (385). Many adaptive processes can vastly reduce the risk of such sickness. Susceptibility to altitude illness varies considerably between individuals, but for a single individual, the symptoms are often reproducible given the same rate of ascent. High-altitude pulmonary odemea is the most important complication of high-altitude illness and its most common cause of death. It typically manifests with 2–4 days of ascent to altitudes above 2,400 m, most commonly beginning on the second night. In the early stage of disease, decreased exercise performance occurs and individuals require increased amount of time to recover from exertions. Individuals also complained of fatigue, weakness, and persistent dry cough, possibly combined with symptoms of acute sickness. As the disease progresses, individuals become short of breath with minimal exertion. Dyspnea at rest, audible chest congestion, generalized pallor, nail bed cyanosis and production of pink frothy sputum are late findings in severe disease. Even in the absence of concurrent high-altitude cerebral edema, severe hypoxemia may produce mental changes, ataxia, and altered levels of consciousness. In general blood gas analysis reveals severe hypoxemia. Pulmonary arterial pressure is high, but pulmonary wedge pressure is normal, and heart size is not increased. Although the mechanism underlying HAPE remains incompletely understood, it appears that the elevated pulmonary artery pressure plays a pivotal role in the process. Multiple studies demonstrated that susceptible individuals have abnormally high pulmonary artery pressure in response to hypoxic breathing, during normoxic and hypoxic exercise, and on high altitude before the onset of edema. Increased sympathetic tone, and alteration in vasoactive mediators-like endothelin-1, NO produced by pulmonary ECs may also lead to stronger hypoxic pulmonary vasoconstriction (384). In autopsies, a red cell rich proteinaceous alveolar exudate with hyaline membrane is characteristic. In all autopsies, areas of pneumonitis with neutrophil accumulation but no evidence of bacterial accumulation has been observed. Most reports mentioned capillary and arterial thrombi, fibrin deposits, hemorrhage, and infarcts. Uneven hypoxic vasoconstriction is discussed. Uneven perfusion is suggested clinically by the typical patchy radiographic appearance and by MRI studies in patients together with hypoxic blood gas parameters which demonstrates greater heterogeneous regional perfusion in HAPE-susceptible subjects (384). The estimated death rate of altitude illness is about 7.7/100,000 trekkers, with increasing mortality during the last decade (386).

Treatment of HAPE consists, if ever possible, in descent from altitude, rest, oxygen supplementation, and administration of drugs such as corticosteroids and furosemide.

Prophylactic inhalation the β2AR agonist salmeterol decreased the HAPE incidence by more than 50% (387). The most pertinent explanation was that salmeterol would enhance the clearance of alveolar fluid since β-adrenergic agonists upregulate the clearance of alveolar fluid by stimulating transepithelial sodium transport. This hypothesis is supported by the fact that the level of sodium transport in the respiratory epithelium is lower in patients susceptible to HAPE. However, the study results cannot exclude the possibility that the beta2 agonist could have modulated vascular permeability or the hemodynamic response associated with hypoxemia and HAPE (4).

In an experimental rat model simulating HAPE by hypobaric and hypoxic conditions equivalent to an altitude of 4,500 m with exhaustive treadmill exercise of 15 m per minute for 24 h, then for an equivalent of altitude of 6,000 m for further 48 h, it has been shown that the TNF tip peptide reduced pulmonary edema and increased the TJ occluding expression compared to high-altitude controls, dexamethasone, and aminophylline treated control animals (7). Compared to untreated high-altitude control animals, TNF tip peptide significantly lowered levels of the inflammatory cytokines TNF, IL-1β, IL-6 and IL-8 in bronchoalveolar lavage. TNF tip peptide-treated animals experienced less pulmonary edema also compared to dexamethasone-treated animals, and was more effective than its comparators in reduction of bronchoalveolar lavage protein content and inflammatory parameters (7). The higher expression of occludin may have translated in an increased stability of the alveolar–capillary barrier, probably related to the reduction in the extent of protein leakage in TNF tip peptide-treated animals. The results suggest that the biologic potential of the TNF tip region is more active in this model than dexamethasone as standard therapy on one hand, and as the glucocorticosteroids (7). The model suggests that HAPE can be treated with TNF tip peptide at least in a part of patients affected, and clinical studies are underway.

However, inhaled budesonide seems not consistently able to prevent acute mountain sickness and HAPE (513).

## Summary and Conclusion

Alveolar fluid reabsorption is of high clinical importance in both cardiac and non-cardiac edema. Clinically, a conservative fluid strategy in ARDS patients resulted in more ventilator-free days (514). There is evidence that lower vascular pressures reduce pro-inflammatory pathways (515), and in chronic hydrostatic pulmonary edema tissue remodeling ensues (516).

Recent studies of cytokine-ion channel interactions have clearly shown that the concept of ion channel modulation to improve AFC has to be broadened, also taking into account previously ignored functions of these mediators. The concept of active interactions between barrier function and ion transporters to maintain lung fluid balance plays a pivotal biological role. TNF’s lectin-like domain, mimicked by the TNF tip peptide, was demonstrated to strengthen capillary barrier function in the presence of bacterial toxins in vitro and in vivo. Indeed, influx, efflux, and tightness of the EC layer are all biologically interrelated. Such a relationship is also present in the alveolar epithelium with interactions with ion transporters and TJs (11). These observations suggest that the biologic potential of ion channel modulation with drugs or peptides is more relevant than initially presumed.

A conceptual problem in ALI and other inflammatory conditions is how fluid reabsorption can function in such an “un-tight system” as in partially destroyed endothelial-interstitial or interstitial-alveolar barriers, and what is the expression level of ion channels in those conditions (25). The same may hold true in the context of hypoxia and the decreased expression of ENaC. Regeneration and repair of injured, apoptotic or necrotic endothelial or AECs can be fostered endogenously by local or bone-marrow derived precursors or by exogenously administered factors, as formerly studied in animal models using progenitor cell populations and stimulants. Clinical refinements are underway and update outcome parameters, such as AFC (517).

In clinical situations with cardiogenic as well as with non-cardiogenic pulmonary edema, i.e., ALI and ARDS, we have to be extremely cautious with prescribing drugs that might interfere with alveolar fluid transports or inflammation. Furosemide might further be the mainstay of diuretic drug and the alveolar flooding stopper especially in cardiogenic edema due to its effect on NKCC1 and CFTR. Amiloride should not be taken. Many clinical questions will be open around beta blocking agents as well as beta stimulating agents in the context of pulmonary edema and will probably depend on their indication. cAMP may play some role, but from which point those two drug classes are counterproductive, remains actually open.

There has been much work focused on one ion channel without considering the interconnection between major biological ion channels or its modulators, which may limit the validity of conclusions or findings of much published work. In future research it would be important to try to better integrate these channels, as well as their interactions with cytokines present in the lung milieu during the various pathologies. Many parallels exist between different organ systems and ion channels, underlining that interdisciplinary network is promising.

As shown in lung transplant primary graft failure, and thus probably also true in ARDS, ALI causes important and systemic long-term injury, especially brain injury. The critical step of high ethical impact for the scientific community is to expand integrative translational research in terms of clinical investigation with the known targets to improve clinical outcome. This is especially important in lung transplantation, as donor shortage still leaves many patients worldwide dying without this therapeutic option, and possibly in ALI and ARDS.

## Author Contributions

All authors significantly contributed to the conceptual work, the writing and editing of the work.

## Conflict of Interest Statement

The authors declare that the research was conducted in the absence of any commercial or financial relationships that could be construed as a potential conflict of interest.
